# Can Quality of Life Assessments Differentiate Heterogeneous Cancer Patients?

**DOI:** 10.1371/journal.pone.0099445

**Published:** 2014-06-11

**Authors:** Ryan M. McCabe, James F. Grutsch, Swetha B. Nutakki, Donald P. Braun, Maurie Markman

**Affiliations:** 1 Medicine & Science, Cancer Treatment Centers of America at Midwestern Regional Medical Center, Zion, Illinois, United States of America; 2 Department of Epidemiology, University of Illinois School of Public Health, Chicago, Illinois, United States of America; 3 Medicine & Science, Cancer Treatment Centers of America at Eastern Regional Medical Center, Philadelphia, Pennsylvania, United States of America; Kyushu University Faculty of Medical Science, Japan

## Abstract

**Purpose:**

This research conducted a face validation study of patient responses to the application of an HRQOL assessment research tool in a comprehensive community cancer program setting across a heterogeneous cohort of cancer patients throughout the natural history of diagnosed malignant disease, many of whom would not be considered candidates for clinical research trial participation.

**Methods:**

Cancer registries at two regional cancer treatment centers identified 11072 cancer patients over a period of nine years. The EORTC QLQ-C30 was administered to patients at the time of their initial clinical presentation to these centers. To determine the significance of differences between patient subgroups, two analytic criteria were used. The Mann-Whitney test was used to determine statistical significance; clinical relevance defined a range of point differences that could be perceived by patients with different health states.

**Results:**

Univariate analyses were conducted across stratification variables for population, disease severity and demographic characteristics. The largest differences were associated with cancer diagnosis and recurrence of disease. Large differences were also found for site of origin, mortality and stage; minimal differences were observed for gender and age. Consistently sensitive QoL scales were appetite loss, fatigue and pain symptoms, and role (work-related), social and physical functions.

**Conclusions:**

1) The EORTC QLQ-C30 collected meaningful patient health assessments in the context of non-research based clinical care, 2) patient assessment differences are manifested disparately across 15 QoL domains, and 3) in addition to indicating how a patient may feel at a point in time, QoL indicators may also reveal information about underlying biological responses to disease progression, treatments, and prospective survival.

## Introduction

Quality of Life (QoL) has become a primary outcome used to measure the value and effectiveness of cancer therapy [1], [2]. Patient reported outcomes may differ from clinician reported observations on symptoms and functioning, and both sets of data when considered together can yield more accurate and predictive patient assessments [3]. Formal QoL assessment tools are not commonly used in clinical practice despite data showing their effectiveness to screen patients for problems, monitor health over time, and improve patient-provider communication [4]–[6]. One problem has been the difficulty in developing guidelines that interpret the clinical significance of differences in QoL scores. The lack of accepted guidelines has delayed oncologists from developing an intuitive grasp of the clinical meaning of assessment scores [4]. QoL assessment tools are in a similar position where blood pressure cuffs were 100 years ago [7]. Today, physicians understand the significance of blood pressure cuff readings to diagnose and manage hypertension in patients. As a result, such measurements are taken in nearly every clinical setting.

Another problem delaying the integration of these tools into routine patient care is the clinicians' lack of confidence that the published results from clinical trials extrapolate to patients undergoing treatment in the community setting [8]. Often times in medicine, patients in the community setting do not experience the outcomes reported in the clinical trial literature. Clinical trials use highly selected and motivated patients undergoing treatment at leading centers; that is, ideal patients being treated under near ideal conditions. Consequently, the current gap in knowledge about what a QoL profile is for a given health state of a patient in the community setting is stalling the incorporation of these tools in routine clinical practice.

This research conducted a face validation of responses to the clinical application of the EORTC QLQ-C30 tool in a heterogeneous cohort of cancer patients throughout the natural history of diagnosed disease, many of whom would not be considered candidates for clinical trial participation. Patients were stratified into subgroups based on clinical and demographic factors known to influence patient longevity in order to identify differences in symptom burden, functioning and overall quality of life between prognostically distinct groups [9], [10]. In contrast to studies that combine results from multiple trials – such as review papers or meta-data analysis applications – data for this research came from a single cohort, collected over nine years and comprising over 11,000 patients from a North American population.

Prior research used anchor-based and evidence-based methods (e.g., meta-analysis) in longitudinal patient data to determine what changes in scale scores were trivial, small, modest and large [4], [11]. Extensive data collected from cross-cultural research settings have shown QoL symptom and functioning scales reported with the EORTC QLQ-C30 instrument can discriminate patients with different performance status [9], [10]. These approaches using either statistical techniques or anchor-based methods have yet to provide a complete mapping of differences in scale scores to clinically different health states [12], [13]. This work used both statistical and clinical significance measures to contextualize for clinicians the magnitudes of differences in QoL scores between clinically distinct patient cohorts (e.g., by stage of tumor, site of origin, relapse, etc.).

## Methods

### Study Design

Research staff offered all prospective patients, regardless of treatment or disease history, an opportunity to complete the EORTC QLQ-C30 instrument upon arrival at Cancer Treatment Centers of America (CTCA) at Southwestern Regional Medical Center in Tulsa, OK or CTCA at Midwestern Regional Medical Center in Zion, IL between January 1, 2001 and December 30, 2009. The only criterion for participation was being able to read and complete the survey in English. The instrument was administered during registration at the patient's first visit before they had an opportunity to visit clinical staff. This research was approved by the CTCA Institutional Review Board.

### QoL Instrument

The EORTC QLQ-C30 is a validated quality of life (QoL) instrument that collects Patient-Reported Outcomes (PROs) [9], [14], [15]. The tool assesses common cancer symptoms querying patient functioning and presence of symptoms. The instrument consists of 30 questions. Responses range from 1 to 4 for symptom and function items (1 = Not at all, 4 = Very much) or 1 to 7 for global health items (1 = Very poor, 7 = Excellent). Responses are linearly transformed to a 0–100 score in each of 15 categorical, non-overlapping scales (i.e., each item response contributes to only one scale score).

There are nine symptom scales. Fatigue, pain, and nausea/vomiting are comprised of multiple items. The remaining symptom scales are composed of single items regarding dyspnea, appetite loss, insomnia, constipation, diarrhea, and perceived financial effects of the disease and treatment. There are five functioning scales: physical (five-questions), role/work-related (two-questions), cognitive (two-questions), emotional (four-questions), and social (two-questions). The global health scale combines responses to two items pertaining to overall quality of life. For functioning scales and global health, a higher score represents better functioning, while for symptoms, a higher score represents greater symptom burden.

### Statistical Methods

Responses were not normally distributed as evident by qualitative visual comparisons and confirmed by a Kolmogorov-Smirnov test comparing distribution to a reference sample (e.g., standard normal distribution). The overall study population was stratified into disparate subgroups for the purpose of making meaningful clinical comparisons. Comparisons were not designed to make cause and effect conclusions but to show differences between archetypical patients representing a variety of clinical conditions. For each set of comparisons, 15 QoL scales which were assumed to be independent were used to test 15 null hypotheses; the multiple comparisons problem was accounted for by a factor of 15 using the Bonferroni correction. Statistical significance was assessed using the Mann-Whitney test with a significance threshold of p<0.05/15 = 0.0033.

### Clinically Relevant Differences

The number of patients in this study and each subgroup comparison was relatively large. Differences between large subgroups, while often statistically significant, do not necessarily indicate clinically meaningful differences in health states. No single set of guidelines exists to categorize differences in patient responses to the QLQ-C30 instrument as small, intermediate, or large differences [4], [16], [17]. QoL investigators have reported that scale scores must differ by a ‘minimal’ level for patients to perceive differences in ability to function [18], [19]. Several reports using the EORTC QLQ-C30 tool found asymmetry in magnitude of clinical differences depending on cancer patients' QoL improving or worsening [20], [21]. It is unclear how these insights apply to an analysis that uses a cross-sectional study design. Anchor-based research on the QoL tool used in this study indicated symmetry in differences and linked changes in QLQ-C30 scale scores to patient perception of differences [11]. This scale was applied herein with clinically relevant differences defined as small (5–10), moderate (10–20) or large (>20). All clinically relevant differences reported were statistically significant, unless noted otherwise.

### Stratification Variables

Stratification variables were chosen by a panel of clinicians considering prognostic power and availability of data. Demographic and clinical data were provided by hospital cancer registries, and all symptom, functioning and global health scales were included for analysis. Patients were included in this study whose disease effects on their longevity ranged from potentially insignificant (e.g., stage 1 & 2 breast cancer) to limiting their life span to months – metastatic pancreatic disease. Patients were stratified by newly diagnosed/recurrent disease, site of origin, mortality, best AJCC (American Joint Committee on Cancer) stage for newly diagnosed patients, re-classified stage for recurrent patients, number of comorbidities (both pre-existing and post-cancer diagnosis), gender, and age at study. Recurrent disease was re-categorized using staging criteria to indicate current disease state. Patients with newly diagnosed and recurrent disease were assessed as separate cohorts for all comparisons. General population (mostly European) values from the EORTC Reference Manual [22] were used to compare cancer patients' responses with those from an undiagnosed population [9].

## Results

### Participant Demographics

This study – conducted from January, 2001 through December, 2009 – identified 23,783 potential participants from which 12,357 agreed to complete the instrument preceding initial clinical consultation. 11,469 patients returned the questionnaire; 397 surveys were disqualified due to incomplete responses leaving 11,072 patients included in this research (46.6% response rate).

Three sub-cohorts of patients responded to the QOL surveys (Table 1): patients with newly diagnosed disease who treated at a participating facility (34.3%); patients with recurrent disease who treated at a participating facility (42.3%); and participants who elected to forego treatment at a participating facility (defined as “consults”, (23.4%)).

**Table 1:** **Characteristics of patients who participated and who chose not to participate in the study.**

Patient Characteristic	Participant Characteristics			Non-Participant Characteristics		
	Newly Diagnosed	Recurrent Disease	Consults	Newly Diagnosed	Recurrent Disease	Consults
N (11072)	3767 (34·0%)	4711 (42·6%)	2594 (23·4%)	3590 (32·9%)	4052 (37·2%)	3256 (29·9%)
Age at study, median	57±10·4	55±10·6	55±11·2	58±11·6	56±11·4	52±12·9
**Sex (%)**						
Male	1834 (48·7%)	1895 (40·2%)	1212 (45·2%)	1779 (49·6%)	1675 (41·3%)	1226 (37·7%)
Female	1933 (51·3%)	2816 (59·8%)	1463 (54·5%)	1811(50·4%)	2377 (58·7%)	1932 (59·3%)
Not Stated	0 (0·0%)	0 (0·0%)	8 (0·3%)	0 (0·0%)	0 (0·0%)	98 (3·0%)
**Site of Origin (%)**						
Lung	730 (19·4%)	682 (14·5%)	335 (12·5%)	599 (16·7%)	609 (15·0%)	388 (11·9%)
Breast	718 (19·1%)	1102 (23·4%)	516 (19·2%)	729 (20·3%)	776 (19·2%)	687(21·1%)
Colorectal	243 (6·5%)	628 (13·3%)	299 (11·1%)	206 (6·7%)	473 (11·7%)	260 (8·0%)
Prostate	527 (14·0%)	285 (6·1%)	241 (9·0%)	642 (17·9%)	275 (6·8%)	247 (7·6%)
Pancreatic	415 (11·0%)	292 (6·2%)	179 (6·7%)	259 (7·2%)	210 (5·2%)	161 (4·9%)
All other Cancers	1134 (30·1%)	1722 (36·6%)	1113 (41·5%)	1155 (32·2%)	1709 (42·2%)	1513 (46·5%)
**Vital Status (%)**						
Alive	2009 (53·3%)	1722 (36·6%)	NA	1717 (47·8%)	1107 (27·3%)	NA
Dead	1758 (46·7%)	2989 (63·4%)	NA	1872 (52·1%)	2945 (72·7%)	NA
**Comorbidities**						
None	1260 (33·5%)	1828 (38·8%)	NA	2160 (60·2%)	2571 (63·5%)	NA
1	687 (18·2%)	729 (15·5%)	NA	537 (15·0%)	480 (11·9%)	NA
2	551 (14·6%)	596 (12·7%)	NA	316 (8·8%)	292 (7·2%)	NA
3 or more	1269 (33·7%)	1558 (33·1%)	NA	577 (16·1%)	709 (17·5%)	NA
**Best AJCC Stage (%)**						
Stage 1	470 (12·5%)	69 (1·4%)^a^	NA	459 (12·8%)	55 (1·4%)^a^	NA
Stage 2	908 (24·1%)	186 (3·8%)^a^	NA	1024 (28·5%)	159 (3·9%)^a^	NA
Stage 3	644 (17·1%)	258 (5·3%)^a^	NA	559 (15·6%)	253 (6·2%)^a^	NA
Stage 4	1434 (38·1%)	3568 (73·5%)^a^	NA	1157 (32·2%)	2789 (68·8%)^a^	NA
Unknown Stage	311 (8·3%)	774 (15·9%)^a^	NA	391 (10·9%)	796 (19·6%)^a^	NA

a patients re-staged following clinical presentation at CTCA.

Median age for patients was 56 years; there was a female preponderance (n = 6374; 55.6%) and 66.3% had disease of the lung, breast, colon, rectum, prostate or pancreas. A significant fraction of patients had newly diagnosed advanced disease (stage 3 or 4, 18.8%) or recurrent disease (42.6%). Most patients who subsequently underwent treatment at a participating facility reported at least one comorbidity (66.5% of newly diagnosed, 61.2% of recurrent disease).

Nearly 23% of the patients who took the EORTC surveys did not undergo therapy at a participating facility. To identify any treatment intention or institutional bias, data were included for the patient and non-patient populations participating in this research (Table 1).

### Eligible Patients Who Did Not Participate

Approximately one-half of eligible patients (11,426) did not respond to the QOL surveys, and 527 non-responders had incomplete demographic data (Table 1). To identify any potential study selection bias, comparison of demographics and clinical variables between participants and non-participants revealed similar distributions of age at study, gender, site of origin, and prevalence of recurrent or advanced disease. An exception was the lower prevalence of comorbidities in non-participants – no comorbidities in 60-63% of non-participants vs. 33-38% for participants.

### Effects of Disease Status and Site of Origin

Patients grouped by site of origin (prostate, breast, colorectal, lung, and pancreatic) were compared between newly diagnosed and recurrent disease (Table 2, Table S10 in File S1). Among the newly diagnosed, subgroups who reported the highest levels of QoL by site of origin reported the largest differences with recurrent disease. For example newly diagnosed prostate patients reported the highest global health and functioning scores and the lowest symptom burden, but on relapse they reported the largest negative differences in scores in global health, physical, role and social functioning, and in symptoms of fatigue, pain, appetite loss and constipation.

**Table 2:** **QoL scale scores and differences between patient sub-groups by site of origin.**

QoL symptoms and functions	Prostate			Breast			Colorectal			Lung			Pancreatic			Others		
	ND (527)	Rec (285)	Diff	ND (718)	Rec (1102)	Diff	ND (243)	Rec (628)	Diff	ND (730)	Rec (682)	Diff	ND (415)	Rec (292)	Diff	ND (1134)	Rec (1722)	Diff
Global Health	72·5	59·9	**12·6_(M)_**	67·0	57·6	**9·4_(S)_**	62·9	57·8	**5·1_(S)_**	55·5	49·5	**6·0_(S)_**	54·0	51·6	**2·4** **	58·9	55·5	**3·4**
Physical Function	88·6	77·7	**10·9_(M)_**	83·9	71·3	**12·6_(M)_**	80·9	74·5	**6·4_(S)_**	72·7	63·7	**9·0_(S)_**	76·6	71·9	**4·7**	76·9	72·1	**4·8**
Role Function	86·1	72·5	**13·6_(M)_**	76·4	64·6	**11·8_(M)_**	68·5	66·6	**1·9** **	61·1	54·6	**6·5_(S)_**	59·8	58·7	**1·1** **	64·7	62·7	**2·0** *
Emotional Function	76·3	71·0	**5·3_(S)_**	64·5	65·6	**−1·1** **	68·7	67·6	**1·1** **	62·1	63·6	**−1·5** **	62·6	66·3	**−3·7** *	64·2	66·7	**−2·5**
Cognitive Function	85·8	78·5	**7·3**	77·6	74·5	**3·1** *	81·1	77·8	**3·3** **	76·1	72·8	**3·3** *	76·5	77·5	**−1·0** **	76·5	76·1	**0·4** **
Social Function	84·5	72·0	**12·5_(M)_**	73·7	63·6	**10·1_(M)_**	70·1	65·0	**5·1_(S)_** *	64·3	56·6	**7·7_(S)_**	58·6	60·4	**−1·8** **	65·5	62·9	**2·6** *
Fatigue	23·0	35·5	**−12·5_(M)_**	33·1	45·0	**−11·9_(M)_**	38·1	43·8	**−5·7_(S)_** *	45·0	52·4	**−7·4_(S)_**	47·9	50·9	**−3·0** **	42·0	45·9	**−3·9**
Nausea/vomiting	4·8	9·8	**−5·0_(S)_** *	8·4	14·7	**−6·3_(S)_**	11·5	14·4	**−2·9** *	12·6	17·5	**−4·9**	20·0	18·4	**1·6** **	13·8	17·2	**−3·4**
Pain	18·7	34·4	**−15·7_(M)_**	26·8	39·0	**−12·2_(M)_**	30·5	36·5	**−6·0_(S)_** *	36·2	42·4	**−6·2_(S)_**	44·6	41·6	**3·0** **	34·7	37·2	**−2·5** *
Dyspnea	12·3	17·9	**−5·6_(S)_**	15·3	26·2	**−10·9_(M)_**	18·2	22·7	**−4·5** **	37·1	41·7	**−4·6** *	19·0	23·1	**−4·1** **	22·2	25·8	**−3·6**
Insomnia	26·7	32·7	**−6·0_(S)_** *	37·4	40·0	**−2·6** **	37·9	38·4	**−0·5** **	42·2	40·6	**1·6** **	41·2	38·5	**2·7** **	39·5	38·4	**1·1** **
Appetite loss	8·8	19·9	**−11·1_(M)_**	18·8	26·6	**−7·8_(S)_**	24·8	28·7	**−3·9** **	29·5	35·6	**−6·1_(S)_** *	43·5	35·4	**8·1_(S)_**	28·7	30·2	**−1·5** **
Constipation	9·7	19·8	**−10·1_(M)_**	16·1	23·3	**−7·2_(S)_**	22·2	20·5	**1·7** **	23·9	26·5	**−2·6** **	32·0	23·4	**8·6_(S)_**	20·8	22·5	**−1·7** **
Diarrhea	7·3	9·5	**−2·2** **	11·0	11·0	**0·0** **	16·9	16·9	**0·0** **	9·2	10·5	**−1·3** **	15·2	17·7	**−2·5** **	11·6	14·9	**−3·3**
Financial	18·4	24·8	**−6·4_(S)_** *	31·0	37·5	**−6·5_(S)_**	31·7	33·7	**−2·0** **	32·5	37·5	**−5·0_(S)_** *	33·6	29·3	**4·3** **	33·0	35·7	**−2·7** *

S, M, L Clinical relevance based on magnitude of point difference (Small:S [5]–[10]; Moderate:M [10]–[20]; Large:L [>20]) (supplementary color Table S10).

**Not Statistically Significant (p>0·05).

*Not Statistically Significant, multiple testing adjusted (p>0·0033).

ND/Rec Newly Diagnosed/Recurrent.

Diff Difference (ND-Rec).

With the exception of prostate and breast patients, there were small differences between the newly diagnosed and recurrent patients by site of origin. Newly diagnosed pancreatic cancer patients reported among the lowest scores for global health and functioning, and the highest symptom burdens. Negligible differences were observed between newly diagnosed and recurrent pancreatic cancer patients. Two unexpected exceptions to this were with appetite loss and constipation, wherein patients with recurrent pancreatic disease reported significantly less severe scores than newly-diagnosed patients (43.5 vs. 35.4, appetite loss; 32.0 vs. 23.4, constipation; newly-diagnosed vs. recurrent disease cohorts, respectively, Table 2, Table S10 in File S1).

### Comparison to Published Reference Values from General Populations

An assumption of the analysis is that the majority of any reference population would be undiagnosed and that cancer patients, across different cultures should report higher symptom and lower functioning scores. Currently, there is no population based reference data from North America. The reference population published by the EORTC [22] was used as the best available comparison (Table S1, Table S9 in File S1). Statistical significance and clinical relevance were applied to these population comparisons for the purpose of providing context.

Newly diagnosed and recurrent patient populations were compared to reported reference values and to each other to differentiate QoL health states at a population level [22], [23]. In comparison to the reference populations, patients reported moderate to large differences for nearly every scale except diarrhea (Table 3, Table S11 in File S1). For both newly diagnosed and recurrent cohorts, the largest differences compared to the general population were financial problems, appetite loss, and social and role (work-related) functioning. A comparison between newly diagnosed and recurrent patients revealed clinically meaningful differences (i.e., lower scores in functioning and higher in symptoms) in global health, physical, role and social functioning, and symptoms of fatigue, pain, and dyspnea.

**Table 3:** **Summary of sub-group comparisons within population, disease severity and demographic characteristics.**

QoL symptoms and functions	Population Characteristics			Disease Severity Characteristics						Demographic Characteristics			
	GP – ND (7802 vs 3767)	GP–Rec (7802 vs 4711)	ND –Rec (3767 vs 4711)	Mortality (> = 3 Months - <3 Months)		Stage (1&2 - 3&4)		Comorbidities (<3 - > = 3)		Gender (Male - Female)		Age (<median - > = median)†	
				ND (3461 vs 304)	Rec (3639 vs 1057)	ND (1378 vs 2074)	Rec (246 vs 3720)	ND (2498 vs 1269)	Rec (3153 vs 1558)	ND (1834 vs 1933)	Rec (1895 vs 2816)	ND (1830 vs 1937)	Rec (2180 vs 2531)
Global Health	9·8_(S)_	15·8_(M)_	6·0_(S)_	14·2_(M)_	10·1_(M)_	9·7_(S)_	6·4_(S)_	8·0_(S)_	8·1_(S)_	0·6**	−1·4*	0·1**	1·4**
Physical Function	10·5_(M)_	18·5_(M)_	8·0_(S)_	13·6_(M)_	10·4_(M)_	8·8_(S)_	8·6_(S)_	6·3_(S)_	5·9_(S)_	3·2	2·9	2·9	2·7
Role Function	15·7_(M)_	21·9_(L)_	6·2_(S)_	18·1_(M)_	13·8_(M)_	14·1_(M)_	11·1_(M)_	8·8_(S)_	5·9_(S)_	0·5**	0·4**	−1·3**	−1·5*
Emotional Function	10·6_(M)_	9·9_(S)_	−0·7**	6·1_(S)_	3·4	4·2	2·3**	5·1_(S)_	4·4	5·3_(S)_	3·4	−6·2_(S)_	−4·1
Cognitive Function	7·9_(S)_	10·4_(M)_	2·5	4·5*	5·4_(S)_	2·3	1·7**	4·0	2·9	4·6	3·4	−3·4	−2·1
Social Function	18·5_(M)_	24·7_(L)_	6·2_(S)_	15·9_(M)_	11·5_(M)_	12·2_(M)_	9·2_(S)_	6·6_(S)_	3·1	3·4	2·5*	−3·0	−3·8
Fatigue	−14·5_(M)_	−21·9_(L)_	−7·4_(S)_	−17·0_(M)_	−12·1_(M)_	−12·6_(M)_	−10·9_(M)_	−8·8_(S)_	−7·2_(S)_	−2·8	−2·4*	1·7*	0·9**
Nausea/vomiting	−8·1_(S)_	−12·2_(M)_	−4·1	−6·1_(S)_	−5·7_(S)_	−6·9_(S)_	−5·0_(S)_	−2·3	−4·0	−1·8	−3·0	2·9	3·5
Pain	−11·2_(M)_	−17·5_(M)_	−6·3_(S)_	−15·5_(M)_	−10·1_(M)_	−8·0_(S)_	−7·4_(S)_	−8·3_(S)_	−5·6_(S)_	−2·2*	−1·8*	4·8	4·2
Dyspnea	−10·0_(M)_	−15·3_(M)_	−5·3_(S)_	−12·9_(M)_	−9·4_(S)_	−8·5_(S)_	−7·5_(S)_	−8·9_(S)_	−5·9_(S)_	−0·9**	0·4**	−3·0*	−1·2**
Insomnia	−16·1_(M)_	−16·9_(M)_	−0·8**	−9·3_(S)_	−4·0	−5·9_(S)_	−5·7_(S)_ *	−6·0_(S)_	−4·7	−2·8*	−2·3*	5·8_(S)_	6·6_(S)_
Appetite loss	−18·9_(M)_	−22·9_(L)_	−4·0	−20·0_(L)_	−11·5_(M)_	−15·0_(M)_	−9·1_(S)_	−8·7_(S)_	−5·8_(S)_	−1·5*	−1·6*	3·0	1·1**
Constipation	−13·6_(M)_	−16·2_(M)_	−2·6	−13.0_(M)_	−8·4_(S)_	−8·6_(S)_	−5·9_(S)_	−5·1_(S)_	−3·9	−2·0*	−3·0	0·2**	2·8*
Diarrhea	−4·2	−6·4_(S)_	−2·2	−3·0*	−1·9*	−1·0**	0·9**	−1·9*	−1·7*	−1·5*	0·7**	2·6*	1·2**
Financial Problems	−21·0_(L)_	−25·6_(L)_	−4·6	−1·1**	−1·0**	−6·2_(S)_	−3·8	−1·1*	−1·9*	−4·3	−3·7	8·4_(S)_	10·1_(M)_

S, M, L Clinical relevance based on magnitude of point difference (Small:S [5]–[10]; Moderate:M [10]–[20]; Large:L [>20]) (supplementary color Table S11).

**Not Statistically Significant (p>0·05).

*Not Statistically Significant, multiple testing adjusted (p>0·0033).

† Median Age for newly diagnosed  = 57 years; Median Age for Recurrent patients  = 55 years.

ND/Rec Newly Diagnosed/Recurrent – all North American – data was collected between 2001–2009.

GP General Population from EORTC reference manual – mostly European– data was collected in the last decade of 20^th^ century.

### Three Month Mortality

Declines in QoL scores have been reported in longitudinal studies as patients approach death [24], [25]. In this study patients were stratified by mortality occurring within three months of survey. Large and moderate, clinically meaningful differences between patient survival sub-groups were seen in global health; role, physical and social function; fatigue, pain, dyspnea, appetite loss, and constipation. These differences were consistent for both newly-diagnosed and recurrent disease cohorts, though differences were larger between newly diagnosed patient subgroups (Table 3, Table S11 in File S1). Interestingly, the mean and median values for recurrent and newly diagnosed patients who died within three months of baseline were similar, and in all scales except appetite loss, mean scores were clinically indistinguishable (Table S2 in File S1).

### Stage

Best AJCC stage was used to classify newly diagnosed patients. Recurrent patients were re-classified by hospital cancer registrars according to standard American College of Surgeons protocols for populating cancer registries. Stage 1 and 2 patients exhibited better global health scores compared to patients with stage 3 or 4 disease for both newly diagnosed and recurrent disease cohorts (Table S3 in File S1). The largest clinically meaningful differences in both cohorts were observed in role, social and physical function and symptoms of appetite loss and fatigue (Table 3, Table S11 in File S1).

### Number of Comorbidities

When patients were stratified between <3 or ≥3 comorbidities, both newly diagnosed and relapsed patient cohorts showed clinically meaningful differences in favor of fewer comorbidities in global health (Table S4 in File S1). Differences favoring fewer comorbidities for newly-diagnosed and recurrent patients were also found for role function and symptoms of fatigue, pain, dyspnea, and appetite loss (Table 3, Table S11 in File S1).

### Gender and Age

Few comparisons across gender or age were statistically significant. Minimal, clinically insignificant differences were found between men and women for both newly diagnosed and recurrent disease patients (Table S5 in File S1). One small difference was observed in newly diagnosed women reporting lower emotional functioning than males.

Patients were stratified into sub-groups of below median age, or equal to and above median age (Table S6 in File S1). In each cohort global health scores were statistically indistinguishable between the two age groups. Three scales showed small differences, disproportionately affecting the younger sub-group: emotional function, insomnia and financial problems (Table 3, Table S11 in File S1).

## Discussion

This study generated a large database of QoL health assessments of heterogeneous cancer patients in a comprehensive community cancer program setting including all phases of the natural history of diagnosed disease. The EORTC QLQ-C30 instrument was found to capture clinically meaningful quality of life differences in patients whose health states ranged from highly curable to hospice bound.

In multiple comparisons moderate and large differences in functioning and symptom scales were found in clinically distinct populations. The largest differences were found when newly diagnosed or relapsed patients were compared to a general population in all scales except diarrhea. A limitation was the lack of availability of a North American reference population.

Clinically meaningful differences were observed when comparing patients categorized by site of origin in newly diagnosed patients. For the global health scale, baseline level differences in descending order were observed for prostate > breast > colorectal > lung > pancreatic and other patients (Table 2, Table S10 in File S1). For certain functional scales, the differences were large enough (∼20 points) to be highly clinically meaningful (e.g., role, social functioning). Similarly, for certain symptom scales, the differences were large enough (20–30 points) to be largely clinically meaningful (e.g., fatigue, pain, appetite loss, constipation). Not surprisingly, dyspnea levels in newly-diagnosed lung cancer patients were highly clinically meaningful and at least twofold greater than levels for other tumor types.

Meaningful differences for newly diagnosed patients could be expected when comparing tumor types with a high preponderance of limited stage disease at diagnosis (e.g., prostate, breast cancer) to tumor types which typically present with more extensive stage disease (e.g., lung, pancreatic). But it is notable that the rank order of functioning and symptom scores was largely maintained when stage 1 and 2 breast and prostate patients were compared to stage 1 and 2 cohorts with colorectal, lung, pancreatic and other cancers (Table S7 in File S1). This suggests that different types of malignant disease that affect QoL are distinct and independent of disease progression, and the tool was able to capture these differences.

Within most sites of origin, relapsed patients reported lower scores for functioning scales and higher symptom scale scores compared to the corresponding newly diagnosed cohort (Table S8 in File S1). Disease progression generally corresponded to clinically significant differences in most functioning and symptom scales. Certain scales – emotional and cognitive functioning, and diarrhea, nausea/vomiting and insomnia – did not reveal differences following disease progression.

Within site of origin subgroups, patients with newly diagnosed prostate or breast disease reported scores that were indistinguishable from the general population's (Table 2, Table S10 in File S1). This was in contrast to newly diagnosed lung and pancreatic cancer patients whose QoL was significantly diminished at diagnosis.

Stage was not found to be a surrogate variable for QoL. Newly diagnosed stage 1 & 2 patients with breast or prostate disease scored nearly identically to the general population, with exceptions of insomnia and financial problems (Table S7 in File S1). Small encapsulated breast and prostate lesions tend not to adversely affect the patient's overall physiological function. However, the emergence of metastatic prostate or breast disease can have an adverse effect on an individual's physiology. This hypothesis could explain the comparatively large negative differences in QoL scales between these patients with stage 1 & 2 and stage 3 & 4 disease [26]. By contrast, newly diagnosed stage 1 & 2 patients in other sites of origin reported moderate to large differences in nearly every scale compared to the general population. This indicates that stage of disease alone is not a sufficient indicator of QoL, and patients with near normal physical assessments may experience mental symptom and functioning burden.

Although most comparison results in this research reflected previous trial research [9], [22], an unexpected observation was made of less severe symptoms of appetite loss and constipation in recurrent pancreatic cancer patients compared with newly diagnosed [27]. This is an example of the potential of this sort of data-driven research conducted across clinical practice to uncover QoL domains that should not be viewed dogmatically in the context of general oncologic practices. Further research is required to show whether such insights can be developed into a tool to identify those pancreatic patients who could benefit from therapy.

Clinically significant differences were observed for multiple function and symptom scales for patients nearing death (mortality < three months from survey). Notably, the magnitudes of these differences were larger for newly diagnosed patients than for those with recurrent disease. When newly diagnosed patients nearing death were compared to recurrent disease patients nearing death, differences across all scales were clinically negligible, except for appetite loss (Table S2 in File S1). This suggests that when patients are stratified by disease progression and near-term mortality, this tool has a greater sensitivity to mortality. This finding supports the hypothesis that patterns of symptom and functioning scale scores could identify patients who are at high risk of dying.

A number of population based survey studies conducted in European populations have reported age and gender differences, but the study cohort showed few differences that were clinically relevant or statistically significant [23], [28].

Certain functioning and symptom scales were found to be relatively more sensitive to differences in a patient's clinical health state. No single scale showed differences across every clinical health state comparison. The symptoms appetite loss, fatigue, pain, and dyspnea were the most consistent and responsive. For patient functioning, physical, role and social functions were most responsive and consistent clinical differentiators. Furthering previous findings by King [13], physical and role functioning scores demonstrated the largest range of means across patient sub-groups, whereas emotional and cognitive function scale means had little variance. Diarrhea, emotional and cognitive scales showed little capacity to differentiate the sub-groups of this study, indicating they may not be effective outcomes measures in this context, regardless of their ability to represent individual patient states.

There were several limitations in this investigation. Clinical relevance of minimally important differences in mean scores was assumed to be the same regardless of direction of difference. This assumption is supported by prior research but is not consistent across all findings [11], [13], [20], [21]. Applying such definitions – derived from longitudinal studies – across different patient sub-groups (e.g., different tumor types) is an additional limitation of this work and may introduce a source of error. Data were not available for every domain relevant to patient QoL, including treatment history, time from diagnosis, performance status and other cancer-specific QoL domains (e.g., peripheral neuropathy). Additional modulating phenomena including response shift [29] and non-disease related factors, such as negative or positive psychological affect, underlying physical robustness, disposition for pain, and the physiological mechanisms for regulating fatigue were also unaccounted [30]–[35]. Although differences in nearly all scales by age and gender were not found, lack of access to primary data from EORTC's reference populations made it difficult to identify methods of adjusting for age and gender distributions; data were reported without adjustment.

Most of the comparisons in this analysis were similar to those found in clinical literature. This was a significant finding because physicians might assume that patients who participate in clinical trials do not fully represent patients undergoing care in community centers. CTCA patient population may be biased because they can travel 500 miles to get treated. Despite these potential biases in the study cohort, the responses to the QLQ-C30 instrument in a clinical setting were similar to the responses recorded in the EORTC research program [22]. This result indicates that the effect of cancer on patients' symptoms, functioning and evaluation of their overall quality of life is relatively independent of the clinical setting (research vs non-research).

There is extensive data showing that the use of QoL tools in the clinic improves patient-provider communication and patient satisfaction with care [36], [37]. The pervasive, everyday use of QoL tools in cancer clinics remains limited for many reasons. The current study provides support for expanding their use in areas relevant to both clinicians and patients. Including QoL patient reported health assessments in routine medical care would catalyze the discovery of relationships among length and quality of life outcomes, including patient symptoms, functioning, global health and survival [38]. It would enable patient differentiation into more personalized clinical subgroups and provide additional support for clinician and patient decision-making.

## Supporting Information

File S1 **Contains the files: Table S1-** Mean, median and standard deviations of QoL attributes for EORTC general population (7802), newly diagnosed (3775) and recurrent disease (4711) patients. **Table S2-** Mean, median and standard deviation of QoL attributes of patients with respect to Mortality < = 3-months Vs >3-months. **Table S3-** Mean, median and standard deviation of QoL attributes of patients with respect to Stage 1&2 vs 3&4. **Table S4-** Mean, median and standard deviation of QoL attributes of patients with respect to Comorbidities <3 vs > = 3. **Table S5-** Mean, median and standard deviation of QoL attributes of patients with respect to Gender and class of case. **Table S6-** Mean, median and standard deviation of QoL attributes of patients with respect to median Age and class of case. **Table S7-** Comparison of mean scores between EORTC published general population and newly diagnosed patients with early stage disease. **Table S8-** Confidence intervals of Patient sub-groups by Site of Origin. **Table S9-** Confidence intervals for EORTC General Population compared with newly diagnosed and recurrent patients. **Table S10-** QoL scale scores and differences between patient sub-groups by site of origin. **Table S11-** Summary of sub-group comparisons within population, disease severity and demographic characteristics.
(ZIP)
