# Supplementary material for: Can Quality of Life Assessments Differentiate Heterogeneous Cancer Patients?
Source: PLoS One. 2014 Jun 11;9(6):e99445. doi: 10.1371/journal.pone.0099445 (PMC4053440; doi:10.1371/journal.pone.0099445)
Supplement: File S1 — Contains the files: Table S1- Mean, median and standard deviations of QoL attributes for EORTC general population (7802), newly diagnosed (3775) and recurrent disease (4711) patients. Table S2- Mean, median and standard deviation of QoL attributes of patients with respect to Mortality < = 3-months Vs >3-months. Table S3- Mean, median and standard deviation of QoL attributes of patients with respect to Stage 1&2 vs 3&4. Table S4- Mean, median and standard deviation of QoL attributes of patients with respect to Comorbidities <3 vs > = 3. Table S5- Mean, median and standard deviation of QoL attributes of patients with respect to Gender and class of case. Table S6- Mean, median and standard deviation of QoL attributes of patients with respect to median Age and class of case. Table S7- Comparison of mean scores between EORTC published general population and newly diagnosed patients with early stage disease. Table S8- Confidence intervals of Patient sub-groups by Site of Origin. Table S9- Confidence intervals for EORTC General Population compared with newly diagnosed and recurrent patients. Table S10- QoL scale scores and differences between patient sub-groups by site of origin. Table S11- Summary of sub-group comparisons within population, disease severity and demographic characteristics. (ZIP) [file pone.0099445.s001.zip › Table S5.docx]

Table S5: Mean, median and standard deviation of QoL attributes of patients with respect to Gender and class of case

| QOL symptoms  and functions | Male newly diagnosed | | | p-values (${\mathrm{Mann}-Whitney test}^{*}$) | CI 95% (±) | Quality of Life Differences | Female newly diagnosed | | | Male recurrent | | | p-values (${\mathrm{Mann}-Whitney test}^{*}$) | CI 95% (±) | Quality of Life Differences | Female recurrent | | |
| --- | --- | --- | --- | --- | --- | --- | --- | --- | --- | --- | --- | --- | --- | --- | --- | --- | --- | --- |
|  | 1834 | | |  |  |  | 1933 | | | 1895 | | |  |  |  | 2816 | | |
|  | Mean | Median | Standard Deviation |  |  |  | Mean | Median | Standard Deviation | Mean | Median | Standard Deviation |  |  |  | Mean | Median | Standard Deviation |
| Global Health | 61.7 | 66.7 | 26.1 | 0.1883 | 1.65 | 0.6 | 61.1 | 66.7 | 25.6 | 54.6 | 58.3 | 26.2 | 0.0343 | 1.51 | 1.4 | 56.0 | 58.3 | 25.7 |
| Physical Function | 80.9 | 86.7 | 21.9 | <0.0001 | 1.43 | 3.2 | 77.7 | 86.7 | 22.9 | 73.1 | 80.0 | 24.4 | <0.0001 | 1.44 | 2.9 | 70.2 | 80.0 | 25.1 |
| Role Function | 69.2 | 83.3 | 33.4 | 0.1491 | 2.10 | 0.5 | 68.7 | 66.7 | 32.5 | 63.1 | 66.7 | 34.0 | 0.2731 | 1.96 | 0.4 | 62.7 | 66.7 | 33.5 |
| Emotional Function | 68.4 | 75.0 | 24.4 | <0.0001 | 1.59 | 5.3 | 63.1 | 66.7 | 25.4 | 68.4 | 75.0 | 24.2 | <0.0001 | 1.44 | 3.4 | 65.0 | 66.7 | 25.1 |
| Cognitive Function | 80.6 | 83.3 | 23.0 | <0.0001 | 1.54 | 4.6 | 76.0 | 83.3 | 25.2 | 77.8 | 83.3 | 24.2 | <0.0001 | 1.46 | 3.4 | 74.4 | 83.3 | 25.6 |
| Social Function | 70.8 | 83.3 | 31.2 | 0.0006 | 2.03 | 3.4 | 67.4 | 66.7 | 32.2 | 64.3 | 66.7 | 32.3 | 0.0066 | 1.89 | 2.5 | 61.8 | 66.7 | 32.7 |
| Fatigue | 37.2 | 33.3 | 28.3 | 0.0008 | 1.79 | 2.8 | 40.0 | 33.3 | 27.9 | 44.6 | 33.3 | 28.1 | 0.008 | 1.66 | 2.4 | 47.0 | 44.4 | 28.9 |
| Nausea/vomiting | 10.9 | 0.0 | 19.5 | 0.0008 | 1.27 | 1.8 | 12.7 | 0.0 | 20.4 | 14.1 | 0.0 | 22.0 | 0.0001 | 1.39 | 3.0 | 17.1 | 0.0 | 24.9 |
| Pain | 30.9 | 16.7 | 30.8 | 0.0096 | 1.98 | 2.2 | 33.1 | 33.3 | 31.2 | 37.3 | 33.3 | 32.5 | 0.0338 | 1.92 | 1.8 | 39.1 | 33.3 | 33.2 |
| Dyspnea | 21.3 | 0.0 | 27.8 | 0.261 | 1.81 | 0.9 | 22.2 | 0.0 | 28.9 | 27.4 | 33.3 | 30.4 | 0.1641 | 1.79 | 0.4 | 27.0 | 33.3 | 31.1 |
| Insomnia | 36.5 | 33.3 | 31.8 | 0.0076 | 2.06 | 2.8 | 39.3 | 33.3 | 32.7 | 37.4 | 33.3 | 32.2 | 0.019 | 1.90 | 2.3 | 39.7 | 33.3 | 33.0 |
| Appetite loss | 24.8 | 0.0 | 31.9 | 0.0417 | 2.04 | 1.5 | 26.3 | 0.0 | 31.9 | 28.7 | 33.3 | 33.5 | 0.0491 | 1.96 | 1.6 | 30.3 | 33.3 | 33.8 |
| Constipation | 19.3 | 0.0 | 28.9 | 0.0155 | 1.87 | 2.0 | 21.3 | 0.0 | 29.5 | 21.1 | 0.0 | 29.7 | 0.0011 | 1.79 | 3.0 | 24.1 | 0.0 | 31.2 |
| Diarrhea | 10.4 | 0.0 | 21.1 | 0.0374 | 1.38 | 1.5 | 11.9 | 0.0 | 22.1 | 13.9 | 0.0 | 23.8 | 0.1581 | 1.37 | 0.7 | 13.2 | 0.0 | 23.4 |
| Financial Problems | 28.3 | 33.3 | 32.4 | 0.0002 | 2.14 | 4.3 | 32.6 | 33.3 | 34.4 | 32.9 | 33.3 | 33.4 | 0.0003 | 1.99 | 3.7 | 36.6 | 33.3 | 34.7 |

* Mann-Whitney test, also known as rank sum test, is a non-parametric test that compares two independent groups.
