# Supplementary material for: Can Quality of Life Assessments Differentiate Heterogeneous Cancer Patients?
Source: PLoS One. 2014 Jun 11;9(6):e99445. doi: 10.1371/journal.pone.0099445 (PMC4053440; doi:10.1371/journal.pone.0099445)
Supplement: File S1 — Contains the files: Table S1- Mean, median and standard deviations of QoL attributes for EORTC general population (7802), newly diagnosed (3775) and recurrent disease (4711) patients. Table S2- Mean, median and standard deviation of QoL attributes of patients with respect to Mortality < = 3-months Vs >3-months. Table S3- Mean, median and standard deviation of QoL attributes of patients with respect to Stage 1&2 vs 3&4. Table S4- Mean, median and standard deviation of QoL attributes of patients with respect to Comorbidities <3 vs > = 3. Table S5- Mean, median and standard deviation of QoL attributes of patients with respect to Gender and class of case. Table S6- Mean, median and standard deviation of QoL attributes of patients with respect to median Age and class of case. Table S7- Comparison of mean scores between EORTC published general population and newly diagnosed patients with early stage disease. Table S8- Confidence intervals of Patient sub-groups by Site of Origin. Table S9- Confidence intervals for EORTC General Population compared with newly diagnosed and recurrent patients. Table S10- QoL scale scores and differences between patient sub-groups by site of origin. Table S11- Summary of sub-group comparisons within population, disease severity and demographic characteristics. (ZIP) [file pone.0099445.s001.zip › Table S4.docx]

Table S4: Mean, median and standard deviation of QoL attributes of patients with respect to Comorbidities <3 vs >=3

| QoL symptoms and functions | Newly diagnosed Comorbidities<3 | | | p-values (${\mathrm{Mann}-Whitney test}^{*}$) |  | Quality of Life Differences | Newly diagnosed Comorbidities>=3 | | | Recurrent Comorbidities<3 | | | p-values (${\mathrm{Mann}-Whitney test}^{*}$) |  | Quality of Life Differences | Recurrent Comorbidities>=3 | | |
| --- | --- | --- | --- | --- | --- | --- | --- | --- | --- | --- | --- | --- | --- | --- | --- | --- | --- | --- |
|  | 2498 | | |  |  |  | 1269 | | | 3153 | | |  |  |  | 1558 | | |
|  | Mean | Median | Standard Deviation |  | CI 95% (±) |  | Mean | Median | Standard Deviation | Mean | Median | Standard Deviation |  | CI 95% (±) |  | Mean | Median | Standard Deviation |
| Global Health | 64.1 | 66.7 | 25.6 | <0.0001 | 1.73 | 8.0 | 56.1 | 58.3 | 25.6 | 58.1 | 58.3 | 25.9 | <0.0001 | 1.50 | 8.1 | 50.0 | 50.0 | 25.0 |
| Physical Function | 81.4 | 86.7 | 21.6 | <0.0001 | 1.51 | 6.3 | 75.1 | 80.0 | 23.6 | 73.3 | 80.0 | 24.4 | <0.0001 | 2.04 | 5.9 | 67.4 | 73.3 | 25.2 |
| Role Function | 71.9 | 83.3 | 31.8 | <0.0001 | 2.21 | 8.8 | 63.1 | 66.7 | 34.2 | 64.8 | 66.7 | 33.1 | <0.0001 | 1.50 | 5.9 | 58.9 | 66.7 | 34.4 |
| Emotional Function | 67.4 | 66.7 | 24.9 | <0.0001 | 1.68 | 5.1 | 62.3 | 66.7 | 25.1 | 67.8 | 66.7 | 24.3 | <0.0001 | 1.52 | 4.4 | 63.4 | 66.7 | 25.6 |
| Cognitive Function | 79.6 | 83.3 | 23.2 | <0.0001 | 1.63 | 4.0 | 75.6 | 83.3 | 25.9 | 76.7 | 83.3 | 24.3 | 0.0021 | 1.97 | 2.9 | 73.8 | 83.3 | 26.6 |
| Social Function | 71.3 | 83.3 | 30.8 | <0.0001 | 2.14 | 6.6 | 64.7 | 66.7 | 33.1 | 63.8 | 66.7 | 32.1 | 0.0025 | 1.72 | 3.1 | 60.7 | 66.7 | 33.4 |
| Fatigue | 35.7 | 33.3 | 27.4 | <0.0001 | 1.88 | 8.8 | 44.5 | 33.3 | 28.6 | 43.7 | 33.3 | 28.6 | <0.0001 | 1.44 | 7.2 | 50.9 | 44.4 | 28.1 |
| Nausea/vomiting | 11.0 | 0.0 | 19.1 | 0.0021 | 1.35 | 2.3 | 13.3 | 0.0 | 21.4 | 14.6 | 0.0 | 22.7 | <0.0001 | 1.99 | 4.0 | 18.6 | 8.3 | 25.8 |
| Pain | 29.3 | 16.7 | 29.8 | <0.0001 | 2.08 | 8.3 | 37.6 | 33.3 | 32.5 | 36.5 | 33.3 | 32.4 | <0.0001 | 1.86 | 5.6 | 42.1 | 33.3 | 33.6 |
| Dyspnea | 18.8 | 0.0 | 26.6 | <0.0001 | 1.90 | 8.9 | 27.7 | 33.3 | 30.7 | 25.2 | 33.3 | 29.9 | <0.0001 | 1.98 | 5.9 | 31.1 | 33.3 | 32.3 |
| Insomnia | 35.9 | 33.3 | 31.6 | <0.0001 | 2.17 | 6.0 | 41.9 | 33.3 | 33.3 | 37.2 | 33.3 | 32.2 | <0.0001 | 2.04 | 4.7 | 41.9 | 33.3 | 33.5 |
| Appetite loss | 22.6 | 0.0 | 30.8 | <0.0001 | 2.14 | 8.7 | 31.3 | 33.3 | 33.3 | 27.7 | 33.3 | 32.7 | <0.0001 | 1.86 | 5.8 | 33.5 | 33.3 | 35.2 |
| Constipation | 18.6 | 0.0 | 28.3 | <0.0001 | 1.97 | 5.1 | 23.7 | 0.0 | 30.8 | 21.6 | 0.0 | 30.1 | <0.0001 | 1.43 | 3.9 | 25.5 | 0.0 | 31.8 |
| Diarrhea | 10.5 | 0.0 | 20.9 | 0.0552 | 1.46 | 1.9 | 12.4 | 0.0 | 23.0 | 12.9 | 0.0 | 23.2 | 0.0115 | 2.08 | 1.7 | 14.6 | 0.0 | 24.1 |
| Financial Problems | 30.1 | 33.3 | 33.1 | 0.3067 | 2.26 | 1.1 | 31.2 | 33.3 | 34.2 | 35.7 | 33.3 | 34.2 | 0.0289 | 1.56 | 1.9 | 33.8 | 33.3 | 34.3 |

* Mann-Whitney test, also known as rank sum test, is a non-parametric test that compares two independent groups.
