# Supplementary material for: Can Quality of Life Assessments Differentiate Heterogeneous Cancer Patients?
Source: PLoS One. 2014 Jun 11;9(6):e99445. doi: 10.1371/journal.pone.0099445 (PMC4053440; doi:10.1371/journal.pone.0099445)
Supplement: File S1 — Contains the files: Table S1- Mean, median and standard deviations of QoL attributes for EORTC general population (7802), newly diagnosed (3775) and recurrent disease (4711) patients. Table S2- Mean, median and standard deviation of QoL attributes of patients with respect to Mortality < = 3-months Vs >3-months. Table S3- Mean, median and standard deviation of QoL attributes of patients with respect to Stage 1&2 vs 3&4. Table S4- Mean, median and standard deviation of QoL attributes of patients with respect to Comorbidities <3 vs > = 3. Table S5- Mean, median and standard deviation of QoL attributes of patients with respect to Gender and class of case. Table S6- Mean, median and standard deviation of QoL attributes of patients with respect to median Age and class of case. Table S7- Comparison of mean scores between EORTC published general population and newly diagnosed patients with early stage disease. Table S8- Confidence intervals of Patient sub-groups by Site of Origin. Table S9- Confidence intervals for EORTC General Population compared with newly diagnosed and recurrent patients. Table S10- QoL scale scores and differences between patient sub-groups by site of origin. Table S11- Summary of sub-group comparisons within population, disease severity and demographic characteristics. (ZIP) [file pone.0099445.s001.zip › Table S3.docx]

Table S3: Mean, median and standard deviation of QoL attributes of patients with respect to Stage 1&2 vs 3&4

| QoL symptoms and functions | Newly diagnosed Stage 1&2 | | | p-values (${\mathrm{Mann}-Whitney test}^{*}$) | CI 95% (±) | Quality of Life Differences | Newly diagnosed Stage 3&4 | | | Recurrent Stage 1&2 | | | p-values (${\mathrm{Mann}-Whitney test}^{*}$) | CI 95% (±) | Quality of Life Differences | Recurrent Stage 3&4 | | |
| --- | --- | --- | --- | --- | --- | --- | --- | --- | --- | --- | --- | --- | --- | --- | --- | --- | --- | --- |
|  | 1378 | | |  |  |  | 2074 | | | 246 | | |  |  |  | 3720 | | |
|  | Mean | Median | Standard Deviation |  |  |  | Mean | Median | Standard Deviation | Mean | Median | Standard Deviation |  |  |  | Mean | Median | Standard Deviation |
| Global Health | 67.6 | 75.0 | 24.6 | <0.0001 | 1.73 | 9.7 | 57.9 | 58.3 | 25.9 | 60.9 | 62.5 | 26.5 | 0.0002 | 3.34 | 6.4 | 54.5 | 58.3 | 25.8 |
| Physical Function | 85.0 | 93.3 | 19.5 | <0.0001 | 1.49 | 8.8 | 76.2 | 80.0 | 23.3 | 79.0 | 86.7 | 22.8 | <0.0001 | 3.21 | 8.6 | 70.4 | 80.0 | 25.0 |
| Role Function | 77.9 | 100.0 | 29.8 | <0.0001 | 2.19 | 14.1 | 63.8 | 66.7 | 33.6 | 72.8 | 83.3 | 32.3 | <0.0001 | 4.35 | 11.1 | 61.7 | 66.7 | 33.8 |
| Emotional Function | 68.4 | 75.0 | 24.8 | <0.0001 | 1.70 | 4.2 | 64.2 | 66.7 | 25.1 | 68.4 | 75.0 | 25.4 | 0.0556 | 3.20 | 2.3 | 66.1 | 66.7 | 24.8 |
| Cognitive Function | 79.8 | 83.3 | 23.7 | 0.0026 | 1.64 | 2.3 | 77.5 | 83.3 | 24.4 | 77.3 | 83.3 | 24.8 | 0.1272 | 3.25 | 1.7 | 75.6 | 83.3 | 25.2 |
| Social Function | 76.7 | 83.3 | 29.7 | <0.0001 | 2.11 | 12.2 | 64.5 | 66.7 | 31.9 | 71.1 | 83.3 | 31.1 | <0.0001 | 4.21 | 9.2 | 61.9 | 66.7 | 32.7 |
| Fatigue | 30.7 | 33.3 | 26.4 | <0.0001 | 1.86 | 12.6 | 43.3 | 33.3 | 27.9 | 36.3 | 33.3 | 27.5 | <0.0001 | 3.67 | 10.9 | 47.2 | 44.4 | 28.5 |
| Nausea/vomiting | 7.6 | 0.0 | 15.5 | <0.0001 | 1.35 | 6.9 | 14.5 | 0.0 | 22.1 | 11.5 | 0.0 | 20.8 | 0.0003 | 3.09 | 5.0 | 16.5 | 0.0 | 24.2 |
| Pain | 24.3 | 16.7 | 27.7 | <0.0001 | 2.06 | 8.0 | 36.3 | 33.3 | 31.9 | 32.2 | 33.3 | 31.0 | 0.0004 | 4.26 | 7.4 | 39.6 | 33.3 | 33.2 |
| Dyspnea | 16.5 | 0.0 | 25.2 | <0.0001 | 1.91 | 8.5 | 25.0 | 33.3 | 29.8 | 20.5 | 0.0 | 27.0 | 0.0003 | 3.98 | 7.5 | 28.0 | 33.3 | 31.1 |
| Insomnia | 34.3 | 33.3 | 31.7 | <0.0001 | 2.19 | 5.9 | 40.2 | 33.3 | 32.5 | 33.5 | 33.3 | 31.9 | 0.0042 | 4.21 | 5.7 | 39.2 | 33.3 | 32.7 |
| Appetite loss | 16.4 | 0.0 | 26.5 | <0.0001 | 2.11 | 15.0 | 31.4 | 33.3 | 33.6 | 21.8 | 0.0 | 30.8 | <0.0001 | 4.36 | 9.1 | 30.9 | 33.3 | 34.0 |
| Constipation | 14.9 | 0.0 | 25.0 | <0.0001 | 1.96 | 8.6 | 23.5 | 0.0 | 30.9 | 17.9 | 0.0 | 29.0 | 0.0015 | 3.99 | 5.9 | 23.8 | 0.0 | 31.1 |
| Diarrhea | 10.7 | 0.0 | 20.9 | 0.2388 | 1.48 | 1.0 | 11.7 | 0.0 | 22.3 | 14.1 | 0.0 | 24.3 | 0.3378 | 3.01 | 0.9 | 13.2 | 0.0 | 23.2 |
| Financial Problems | 26.6 | 0.0 | 32.7 | <0.0001 | 2.28 | 6.2 | 32.8 | 33.3 | 33.8 | 31.0 | 33.3 | 33.5 | 0.0513 | 4.42 | 3.8 | 34.8 | 33.3 | 34.3 |

* Mann-Whitney test, also known as rank sum test, is a non-parametric test that compares two independent groups.
