# Supplementary material for: Can Quality of Life Assessments Differentiate Heterogeneous Cancer Patients?
Source: PLoS One. 2014 Jun 11;9(6):e99445. doi: 10.1371/journal.pone.0099445 (PMC4053440; doi:10.1371/journal.pone.0099445)
Supplement: File S1 — Contains the files: Table S1- Mean, median and standard deviations of QoL attributes for EORTC general population (7802), newly diagnosed (3775) and recurrent disease (4711) patients. Table S2- Mean, median and standard deviation of QoL attributes of patients with respect to Mortality < = 3-months Vs >3-months. Table S3- Mean, median and standard deviation of QoL attributes of patients with respect to Stage 1&2 vs 3&4. Table S4- Mean, median and standard deviation of QoL attributes of patients with respect to Comorbidities <3 vs > = 3. Table S5- Mean, median and standard deviation of QoL attributes of patients with respect to Gender and class of case. Table S6- Mean, median and standard deviation of QoL attributes of patients with respect to median Age and class of case. Table S7- Comparison of mean scores between EORTC published general population and newly diagnosed patients with early stage disease. Table S8- Confidence intervals of Patient sub-groups by Site of Origin. Table S9- Confidence intervals for EORTC General Population compared with newly diagnosed and recurrent patients. Table S10- QoL scale scores and differences between patient sub-groups by site of origin. Table S11- Summary of sub-group comparisons within population, disease severity and demographic characteristics. (ZIP) [file pone.0099445.s001.zip › Table S2.docx]

Table S2: Mean, median and standard deviation of QoL attributes of patients with respect to Mortality <= 3-months Vs > 3-months

| QoL symptoms and functions | Newly diagnosed Mortality <=3 | | | p-values (${\mathrm{Mann}-Whitney test}^{*}$) | CI 95% (±) | Quality of Life Differences | Newly diagnosed Mortality >3 | | | Recurrent Mortality <=3 | | | p-values (${\mathrm{Mann}-Whitney test}^{*}$) | CI 95% (±) | Quality of Life Differences | Recurrent  Mortality >3 | | |
| --- | --- | --- | --- | --- | --- | --- | --- | --- | --- | --- | --- | --- | --- | --- | --- | --- | --- | --- |
|  | 304 | | |  |  |  | 3461 | | | 1057 | | |  |  |  | 3639 | | |
|  | Mean | Median | Standard Deviation |  |  |  | Mean | Median | Standard Deviation | Mean | Median | Standard Deviation |  |  |  | Mean | Median | Standard Deviation |
| Global Health | 48.3 | 50.0 | 26.9 | <0.0001 | 3.00 | -14.2 | 62.5 | 66.7 | 25.4 | 47.6 | 50.0 | 26.7 | <0.0001 | 1.75 | -10.1 | 57.7 | 58.3 | 25.2 |
| Physical Function | 66.8 | 73.3 | 26.1 | <0.0001 | 2.60 | -13.6 | 80.4 | 86.7 | 21.8 | 63.3 | 66.7 | 26.9 | <0.0001 | 1.67 | -10.4 | 73.7 | 80.0 | 23.7 |
| Role Function | 52.3 | 66.7 | 36.1 | <0.0001 | 3.82 | -18.1 | 70.4 | 83.3 | 32.2 | 52.1 | 50.0 | 34.8 | <0.0001 | 2.27 | -13.8 | 65.9 | 66.7 | 32.8 |
| Emotional Function | 60.1 | 66.7 | 27.1 | 0.0001 | 2.93 | -6.1 | 66.2 | 66.7 | 24.8 | 63.7 | 66.7 | 25.4 | <0.0001 | 1.70 | -3.4 | 67.1 | 66.7 | 24.6 |
| Cognitive Function | 74.1 | 83.3 | 27.2 | 0.0071 | 2.84 | -4.5 | 78.6 | 83.3 | 23.9 | 71.5 | 83.3 | 28.0 | <0.0001 | 1.72 | -5.4 | 76.9 | 83.3 | 24.1 |
| Social Function | 54.5 | 66.7 | 34.4 | <0.0001 | 3.69 | -15.9 | 70.4 | 66.7 | 31.2 | 53.9 | 66.7 | 33.8 | <0.0001 | 2.20 | -11.5 | 65.4 | 66.7 | 31.7 |
| Fatigue | 54.2 | 55.6 | 30.1 | <0.0001 | 3.25 | 17.0 | 37.2 | 33.3 | 27.5 | 55.4 | 55.6 | 29.7 | <0.0001 | 1.93 | 12.1 | 43.3 | 33.3 | 27.8 |
| Nausea/vomiting | 17.4 | 0.0 | 24.3 | <0.0001 | 2.33 | 6.1 | 11.3 | 0.0 | 19.5 | 20.3 | 16.7 | 26.8 | <0.0001 | 1.63 | 5.7 | 14.6 | 0.0 | 22.8 |
| Pain | 46.3 | 41.7 | 33.6 | <0.0001 | 3.60 | 15.5 | 30.8 | 16.7 | 30.4 | 46.2 | 33.3 | 34.6 | <0.0001 | 2.24 | 10.1 | 36.1 | 33.3 | 32.1 |
| Dyspnea | 33.6 | 33.3 | 34.1 | <0.0001 | 3.30 | 12.9 | 20.7 | 0.0 | 27.6 | 34.5 | 33.3 | 33.7 | <0.0001 | 2.10 | 9.4 | 25.1 | 33.3 | 29.6 |
| Insomnia | 46.5 | 33.3 | 33.1 | <0.0001 | 3.77 | 9.3 | 37.2 | 33.3 | 32.1 | 41.8 | 33.3 | 33.8 | 0.0010 | 2.24 | 4.0 | 37.8 | 33.3 | 32.3 |
| Appetite loss | 44.0 | 33.3 | 36.9 | <0.0001 | 3.69 | 20.0 | 24.0 | 0.0 | 30.9 | 38.5 | 33.3 | 36.4 | <0.0001 | 2.28 | 11.5 | 27.0 | 33.3 | 32.4 |
| Constipation | 32.2 | 33.3 | 34.2 | <0.0001 | 3.40 | 13.0 | 19.2 | 0.0 | 28.5 | 29.4 | 33.3 | 34.1 | <0.0001 | 2.09 | 8.4 | 21.0 | 0.0 | 29.4 |
| Diarrhea | 13.9 | 0.0 | 24.2 | 0.0440 | 2.54 | 3.0 | 10.9 | 0.0 | 21.4 | 14.9 | 0.0 | 25.3 | 0.0888 | 1.61 | 1.9 | 13.0 | 0.0 | 23.0 |
| Financial Problems | 31.5 | 33.3 | 33.9 | 0.3131 | 3.93 | 1.1 | 30.4 | 33.3 | 33.5 | 35.9 | 33.3 | 34.8 | 0.2661 | 2.34 | 1.0 | 34.9 | 33.3 | 34.1 |

* Mann-Whitney test, also known as rank sum test, is a non-parametric test that compares two independent groups.
