# Supplementary material for: Can Quality of Life Assessments Differentiate Heterogeneous Cancer Patients?
Source: PLoS One. 2014 Jun 11;9(6):e99445. doi: 10.1371/journal.pone.0099445 (PMC4053440; doi:10.1371/journal.pone.0099445)
Supplement: File S1 — Contains the files: Table S1- Mean, median and standard deviations of QoL attributes for EORTC general population (7802), newly diagnosed (3775) and recurrent disease (4711) patients. Table S2- Mean, median and standard deviation of QoL attributes of patients with respect to Mortality < = 3-months Vs >3-months. Table S3- Mean, median and standard deviation of QoL attributes of patients with respect to Stage 1&2 vs 3&4. Table S4- Mean, median and standard deviation of QoL attributes of patients with respect to Comorbidities <3 vs > = 3. Table S5- Mean, median and standard deviation of QoL attributes of patients with respect to Gender and class of case. Table S6- Mean, median and standard deviation of QoL attributes of patients with respect to median Age and class of case. Table S7- Comparison of mean scores between EORTC published general population and newly diagnosed patients with early stage disease. Table S8- Confidence intervals of Patient sub-groups by Site of Origin. Table S9- Confidence intervals for EORTC General Population compared with newly diagnosed and recurrent patients. Table S10- QoL scale scores and differences between patient sub-groups by site of origin. Table S11- Summary of sub-group comparisons within population, disease severity and demographic characteristics. (ZIP) [file pone.0099445.s001.zip › Table S1.docx]

Table S1: Mean, median and standard deviations of QoL attributes for EORTC general population (7802), newly diagnosed (3775) and recurrent disease (4711) patients

| QoL symptoms and functions | EORTC general population | | | CTCA Newly Diagnosed patients | | | Newly Diagnosed vs Recurrent | | | CTCA Recurrent disease population | | | CTCA Consults | | |
| --- | --- | --- | --- | --- | --- | --- | --- | --- | --- | --- | --- | --- | --- | --- | --- |
|  | 7802 | | | 3767 | | |  |  |  | 4711 | | | 2594 | | |
|  | Mean | Median | Standard Deviation | Mean | Median | Standard Deviation | p-values (Mann-Whitney test*) | Quality of Life Differences | CI 95% (±) | Mean | Median | Standard Deviation | Mean | Median | Standard Deviation |
| Global Health | 71.2 | 75.0 | 22.4 | 61.4 | 66.7 | 25.9 | <0.0001 | 6.0 | 1.11 | 55.4 | 58.3 | 25.9 | 57.0 | 58.3 | 26.5 |
| Physical Function | 89.8 | 100.0 | 16.2 | 79.3 | 86.7 | 22.5 | <0.0001 | 8.0 | 1.02 | 71.3 | 80.0 | 24.8 | 73.2 | 80.0 | 25.4 |
| Role Function | 84.7 | 100.0 | 25.4 | 69.0 | 66.7 | 32.9 | <0.0001 | 6.2 | 1.43 | 62.8 | 66.7 | 33.7 | 65.0 | 66.7 | 33.3 |
| Emotional Function | 76.3 | 83.3 | 22.8 | 65.7 | 66.7 | 25.0 | 0.1018 | 0.7 | 1.07 | 66.4 | 66.7 | 24.8 | 65.9 | 66.7 | 25.0 |
| Cognitive Function | 86.1 | 100.0 | 20.0 | 78.2 | 83.3 | 24.2 | <0.0001 | 2.5 | 1.06 | 75.7 | 83.3 | 25.1 | 75.6 | 83.3 | 25.2 |
| Social Function | 87.5 | 100.0 | 22.9 | 69.0 | 66.7 | 31.8 | <0.0001 | 6.2 | 1.38 | 62.8 | 66.7 | 32.5 | 65.0 | 66.7 | 32.0 |
| Fatigue | 24.1 | 22.2 | 24.0 | 38.6 | 33.3 | 28.1 | <0.0001 | 7.4 | 1.22 | 46.0 | 44.4 | 28.6 | 44.1 | 33.3 | 29.0 |
| Nausea/vomiting | 3.7 | 0.0 | 11.7 | 11.8 | 0.0 | 20.0 | <0.0001 | 4.1 | 0.95 | 15.9 | 0.0 | 23.8 | 14.9 | 0.0 | 23.1 |
| Pain | 20.9 | 0.0 | 27.6 | 32.1 | 16.7 | 31.0 | <0.0001 | 6.3 | 1.38 | 38.4 | 33.3 | 32.9 | 35.1 | 33.3 | 32.3 |
| Dyspnea | 11.8 | 0.0 | 22.8 | 21.8 | 0.0 | 28.4 | <0.0001 | 5.3 | 1.27 | 27.1 | 33.3 | 30.8 | 25.4 | 33.3 | 30.1 |
| Insomnia | 21.8 | 0.0 | 29.7 | 37.9 | 33.3 | 32.3 | 0.1602 | 0.8 | 1.39 | 38.7 | 33.3 | 32.7 | 38.5 | 33.3 | 33.8 |
| Appetite loss | 6.7 | 0.0 | 18.3 | 25.6 | 0.0 | 31.9 | <0.0001 | 4.0 | 1.41 | 29.6 | 33.3 | 33.7 | 27.7 | 33.3 | 33.2 |
| Constipation | 6.7 | 0.0 | 18.4 | 20.3 | 0.0 | 29.2 | <0.0001 | 2.6 | 1.29 | 22.9 | 0.0 | 30.7 | 20.8 | 0.0 | 29.3 |
| Diarrhea | 7.0 | 0.0 | 18.0 | 11.2 | 0.0 | 21.6 | <0.0001 | 2.2 | 0.97 | 13.4 | 0.0 | 23.5 | 12.7 | 0.0 | 23.3 |
| Financial Problems | 9.5 | 0.0 | 23.3 | 30.5 | 33.3 | 33.5 | <0.0001 | 4.6 | 1.45 | 35.1 | 33.3 | 34.2 | 34.4 | 33.3 | 34.8 |

* Mann-Whitney test, also known as rank sum test, is a non-parametric test that compares two independent groups.
