# Supplementary material for: Can Quality of Life Assessments Differentiate Heterogeneous Cancer Patients?
Source: PLoS One. 2014 Jun 11;9(6):e99445. doi: 10.1371/journal.pone.0099445 (PMC4053440; doi:10.1371/journal.pone.0099445)
Supplement: File S1 — Contains the files: Table S1- Mean, median and standard deviations of QoL attributes for EORTC general population (7802), newly diagnosed (3775) and recurrent disease (4711) patients. Table S2- Mean, median and standard deviation of QoL attributes of patients with respect to Mortality < = 3-months Vs >3-months. Table S3- Mean, median and standard deviation of QoL attributes of patients with respect to Stage 1&2 vs 3&4. Table S4- Mean, median and standard deviation of QoL attributes of patients with respect to Comorbidities <3 vs > = 3. Table S5- Mean, median and standard deviation of QoL attributes of patients with respect to Gender and class of case. Table S6- Mean, median and standard deviation of QoL attributes of patients with respect to median Age and class of case. Table S7- Comparison of mean scores between EORTC published general population and newly diagnosed patients with early stage disease. Table S8- Confidence intervals of Patient sub-groups by Site of Origin. Table S9- Confidence intervals for EORTC General Population compared with newly diagnosed and recurrent patients. Table S10- QoL scale scores and differences between patient sub-groups by site of origin. Table S11- Summary of sub-group comparisons within population, disease severity and demographic characteristics. (ZIP) [file pone.0099445.s001.zip › Table S11.docx]

Table S11: Summary of sub-group comparisons within population, disease severity and demographic characteristics.

| **QoL symptoms and functions** | **Population Characteristics** | | | **Disease Severity Characteristics** | | | | | | **Demographic Characteristics** | | | |
| --- | --- | --- | --- | --- | --- | --- | --- | --- | --- | --- | --- | --- | --- |
|  | **GP – ND (7802 vs 3767)** | **GP–Rec (7802 vs 4711)** | **ND –Rec (3767 vs 4711)** | **Mortality**  **(>=3 Months - <3 Months)** | | **Stage**  **(1&2 - 3&4)** | | **Comorbidities**  **(<3 - >=3)** | | **Gender**  **(Male - Female)** | | **Age**  **(<median - >=median)^†^** | |
|  |  |  |  | **ND (3461 vs 304)** | **Rec (3639 vs 1057)** | **ND (1378 vs 2074)** | **Rec (246 vs 3720)** | **ND (2498 vs 1269)** | **Rec (3153 vs 1558)** | **ND (1834 vs 1933)** | **Rec (1895 vs 2816)** | **ND (1830 vs 1937)** | **Rec (2180 vs 2531)** |
| Global Health | 9·8 | 15·8 | 6·0 | 14·2 | 10·1 | 9·7 | 6·4 | 8·0 | 8·1 | 0·6^**^ | -1·4**^*^** | 0·1^**^ | 1·4^**^ |
| Physical Function | 10·5 | 18·5 | 8·0 | 13·6 | 10·4 | 8·8 | 8·6 | 6·3 | 5·9 | 3·2 | 2·9 | 2·9 | 2·7 |
| Role Function | 15·7 | 21·9 | 6·2 | 18·1 | 13·8 | 14·1 | 11·1 | 8·8 | 5·9 | 0·5^**^ | 0·4^**^ | -1·3^**^ | -1·5**^*^** |
| Emotional Function | 10·6 | 9·9 | -0·7^**^ | 6·1 | 3·4 | 4·2 | 2·3^**^ | 5·1 | 4·4 | 5·3 | 3·4 | -6·2 | -4·1 |
| Cognitive Function | 7·9 | 10·4 | 2·5 | 4·5**^*^** | 5·4 | 2·3 | 1·7^**^ | 4·0 | 2·9 | 4·6 | 3·4 | -3·4 | -2·1 |
| Social Function | 18·5 | 24·7 | 6·2 | 15·9 | 11·5 | 12·2 | 9·2 | 6·6 | 3·1 | 3·4 | 2·5**^*^** | -3·0 | -3·8 |
| Fatigue | -14·5 | -21·9 | -7·4 | -17·0 | -12·1 | -12·6 | -10·9 | -8·8 | -7·2 | -2·8 | -2·4**^*^** | 1·7**^*^** | 0·9^**^ |
| Nausea/vomiting | -8·1 | -12·2 | -4·1 | -6·1 | -5·7 | -6·9 | -5·0 | -2·3 | -4·0 | -1·8 | -3·0 | 2·9 | 3·5 |
| Pain | -11·2 | -17·5 | -6·3 | -15·5 | -10·1 | -8·0 | -7·4 | -8·3 | -5·6 | -2·2**^*^** | -1·8**^*^** | 4·8 | 4·2 |
| Dyspnea | -10·0 | -15·3 | -5·3 | -12·9 | -9·4 | -8·5 | -7·5 | -8·9 | -5·9 | -0·9^**^ | 0·4^**^ | -3·0**^*^** | -1·2^**^ |
| Insomnia | -16·1 | -16·9 | -0·8^**^ | -9·3 | -4·0 | -5·9 | -5·7**^*^** | -6·0 | -4·7 | -2·8**^*^** | -2·3**^*^** | 5·8 | 6·6 |
| Appetite loss | -18·9 | -22·9 | -4·0 | -20·0 | -11·5 | -15·0 | -9·1 | -8·7 | -5·8 | -1·5**^*^** | -1·6**^*^** | 3·0 | 1·1^**^ |
| Constipation | -13·6 | -16·2 | -2·6 | -13 | -8·4 | -8·6 | -5·9 | -5·1 | -3·9 | -2·0**^*^** | -3·0 | 0·2^**^ | 2·8**^*^** |
| Diarrhea | -4·2 | -6·4 | -2·2 | -3·0**^*^** | -1·9**^*^** | -1·0^**^ | 0·9^**^ | -1·9**^*^** | -1·7**^*^** | -1·5**^*^** | 0·7^**^ | 2·6**^*^** | 1·2^**^ |
| Financial Problems | -21·0 | -25·6 | -4·6 | -1·1^**^ | -1·0^**^ | -6·2 | -3·8 | -1·1**^*^** | -1·9**^*^** | -4·3 | -3·7 | 8·4 | 10·1 |


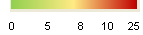
 Clinical relevance based on magnitude of point difference (Small: 5-10, Moderate: 10-20, Large: >20)

** Not Statistically Significant (p>0·05)

* Not Statistically Significant, multiple testing adjusted (p>0·0033)

† Median Age for newly diagnosed = 57 years; Median Age for Recurrent patients = 55 years

ND/Rec Newly Diagnosed/Recurrent – all North American – data was collected between 2001-2009

GP General Population from EORTC reference manual – mostly European– data was collected in the last decade of 20^th^ century
