# Supplementary material for: Can Quality of Life Assessments Differentiate Heterogeneous Cancer Patients?
Source: PLoS One. 2014 Jun 11;9(6):e99445. doi: 10.1371/journal.pone.0099445 (PMC4053440; doi:10.1371/journal.pone.0099445)
Supplement: File S1 — Contains the files: Table S1- Mean, median and standard deviations of QoL attributes for EORTC general population (7802), newly diagnosed (3775) and recurrent disease (4711) patients. Table S2- Mean, median and standard deviation of QoL attributes of patients with respect to Mortality < = 3-months Vs >3-months. Table S3- Mean, median and standard deviation of QoL attributes of patients with respect to Stage 1&2 vs 3&4. Table S4- Mean, median and standard deviation of QoL attributes of patients with respect to Comorbidities <3 vs > = 3. Table S5- Mean, median and standard deviation of QoL attributes of patients with respect to Gender and class of case. Table S6- Mean, median and standard deviation of QoL attributes of patients with respect to median Age and class of case. Table S7- Comparison of mean scores between EORTC published general population and newly diagnosed patients with early stage disease. Table S8- Confidence intervals of Patient sub-groups by Site of Origin. Table S9- Confidence intervals for EORTC General Population compared with newly diagnosed and recurrent patients. Table S10- QoL scale scores and differences between patient sub-groups by site of origin. Table S11- Summary of sub-group comparisons within population, disease severity and demographic characteristics. (ZIP) [file pone.0099445.s001.zip › Table S10.docx]

Table S10: QoL scale scores and differences between patient sub-groups by site of origin.

| **QoL symptoms**  **and functions** | **Prostate** | | | **Breast** | | | **Colorectal** | | | **Lung** | | | **Pancreatic** | | | **Others** | | |
| --- | --- | --- | --- | --- | --- | --- | --- | --- | --- | --- | --- | --- | --- | --- | --- | --- | --- | --- |
|  | **ND (527)** | **Rec (285)** | **Diff** | **ND (718)** | **Rec (1102)** | **Diff** | **ND (243)** | **Rec (628)** | **Diff** | **ND (730)** | **Rec (682)** | **Diff** | **ND (415)** | **Rec (292)** | **Diff** | **ND (1134)** | **Rec (1722)** | **Diff** |
| Global Health | 72·5 | 59·9 | 12·6 | 67·0 | 57·6 | 9·4 | 62·9 | 57·8 | 5·1 | 55·5 | 49·5 | 6·0 | 54·0 | 51·6 | 2·4^**^ | 58·9 | 55·5 | 3·4 |
| Physical Function | 88·6 | 77·7 | 10·9 | 83·9 | 71·3 | 12·6 | 80·9 | 74·5 | 6·4 | 72·7 | 63·7 | 9·0 | 76·6 | 71·9 | 4·7 | 76·9 | 72·1 | 4·8 |
| Role Function | 86·1 | 72·5 | 13·6 | 76·4 | 64·6 | 11·8 | 68·5 | 66·6 | 1·9^**^ | 61·1 | 54·6 | 6·5 | 59·8 | 58·7 | 1·1^**^ | 64·7 | 62·7 | 2·0**^*^** |
| Emotional Function | 76·3 | 71·0 | 5·3 | 64·5 | 65·6 | -1·1^**^ | 68·7 | 67·6 | 1·1^**^ | 62·1 | 63·6 | -1·5^**^ | 62·6 | 66·3 | -3·7**^*^** | 64·2 | 66·7 | -2·5 |
| Cognitive Function | 85·8 | 78·5 | 7·3 | 77·6 | 74·5 | 3·1**^*^** | 81·1 | 77·8 | 3·3^**^ | 76·1 | 72·8 | 3·3**^*^** | 76·5 | 77·5 | -1·0^**^ | 76·5 | 76·1 | 0·4^**^ |
| Social Function | 84·5 | 72·0 | 12·5 | 73·7 | 63·6 | 10·1 | 70·1 | 65·0 | 5·1**^*^** | 64·3 | 56·6 | 7·7 | 58·6 | 60·4 | -1·8^**^ | 65·5 | 62·9 | 2·6**^*^** |
| Fatigue | 23·0 | 35·5 | -12·5 | 33·1 | 45·0 | -11·9 | 38·1 | 43·8 | -5·7**^*^** | 45·0 | 52·4 | -7·4 | 47·9 | 50·9 | -3·0^**^ | 42·0 | 45·9 | -3·9 |
| Nausea/vomiting | 4·8 | 9·8 | -5·0**^*^** | 8·4 | 14·7 | -6·3 | 11·5 | 14·4 | -2·9**^*^** | 12·6 | 17·5 | -4·9 | 20·0 | 18·4 | 1·6^**^ | 13·8 | 17·2 | -3·4 |
| Pain | 18·7 | 34·4 | -15·7 | 26·8 | 39·0 | -12·2 | 30·5 | 36·5 | -6·0**^*^** | 36·2 | 42·4 | -6·2 | 44·6 | 41·6 | 3·0^**^ | 34·7 | 37·2 | -2·5**^*^** |
| Dyspnea | 12·3 | 17·9 | -5·6 | 15·3 | 26·2 | -10·9 | 18·2 | 22·7 | -4·5^**^ | 37·1 | 41·7 | -4·6**^*^** | 19·0 | 23·1 | -4·1^**^ | 22·2 | 25·8 | -3·6 |
| Insomnia | 26·7 | 32·7 | -6·0**^*^** | 37·4 | 40·0 | -2·6^**^ | 37·9 | 38·4 | -0·5^**^ | 42·2 | 40·6 | 1·6^**^ | 41·2 | 38·5 | 2·7^**^ | 39·5 | 38·4 | 1·1^**^ |
| Appetite loss | 8·8 | 19·9 | -11·1 | 18·8 | 26·6 | -7·8 | 24·8 | 28·7 | -3·9^**^ | 29·5 | 35·6 | -6·1**^*^** | 43·5 | 35·4 | 8·1 | 28·7 | 30·2 | -1·5^**^ |
| Constipation | 9·7 | 19·8 | -10·1 | 16·1 | 23·3 | -7·2 | 22·2 | 20·5 | 1·7^**^ | 23·9 | 26·5 | -2·6^**^ | 32·0 | 23·4 | 8·6 | 20·8 | 22·5 | -1·7^**^ |
| Diarrhea | 7·3 | 9·5 | -2·2^**^ | 11·0 | 11·0 | 0·0^**^ | 16·9 | 16·9 | 0·0^**^ | 9·2 | 10·5 | -1·3^**^ | 15·2 | 17·7 | -2·5^**^ | 11·6 | 14·9 | -3·3 |
| Financial | 18·4 | 24·8 | -6·4**^*^** | 31·0 | 37·5 | -6·5 | 31·7 | 33·7 | -2·0^**^ | 32·5 | 37·5 | -5·0**^*^** | 33·6 | 29·3 | 4·3^**^ | 33·0 | 35·7 | -2·7**^*^** |


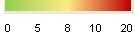
 Clinical relevance based on magnitude of point difference (Small: 5-10, Moderate: 10-20, Large: >20)

** Not Statistically Significant (p>0·05)

* Not Statistically Significant, multiple testing adjusted (p>0·0033)

ND/Rec Newly Diagnosed/Recurrent

Diff Difference (ND-Rec)
