# Supplementary material for: Can Quality of Life Assessments Differentiate Heterogeneous Cancer Patients?
Source: PLoS One. 2014 Jun 11;9(6):e99445. doi: 10.1371/journal.pone.0099445 (PMC4053440; doi:10.1371/journal.pone.0099445)
Supplement: File S1 — Contains the files: Table S1- Mean, median and standard deviations of QoL attributes for EORTC general population (7802), newly diagnosed (3775) and recurrent disease (4711) patients. Table S2- Mean, median and standard deviation of QoL attributes of patients with respect to Mortality < = 3-months Vs >3-months. Table S3- Mean, median and standard deviation of QoL attributes of patients with respect to Stage 1&2 vs 3&4. Table S4- Mean, median and standard deviation of QoL attributes of patients with respect to Comorbidities <3 vs > = 3. Table S5- Mean, median and standard deviation of QoL attributes of patients with respect to Gender and class of case. Table S6- Mean, median and standard deviation of QoL attributes of patients with respect to median Age and class of case. Table S7- Comparison of mean scores between EORTC published general population and newly diagnosed patients with early stage disease. Table S8- Confidence intervals of Patient sub-groups by Site of Origin. Table S9- Confidence intervals for EORTC General Population compared with newly diagnosed and recurrent patients. Table S10- QoL scale scores and differences between patient sub-groups by site of origin. Table S11- Summary of sub-group comparisons within population, disease severity and demographic characteristics. (ZIP) [file pone.0099445.s001.zip › Table S9.docx]

Table S9: Confidence intervals for EORTC General Population compared with newly diagnosed and recurrent patients

| QOL symptoms  and functions | GP-ND | | GP-Rec | |
| --- | --- | --- | --- | --- |
|  | CI 95% (±) | QoL Diff | CI 95% (±) | QoL Diff |
| Global Health | 0.97 | 9·8 | 0.90 | 15·8 |
| Physical Function | 0.87 | 10·5 | 0.87 | 18·5 |
| Role Function | 1.11 | 15·7 | 1.03 | 21·9 |
| Emotional Function | 0.98 | 10·6 | 0.91 | 9·9 |
| Cognitive Function | 0.92 | 7·9 | 0.88 | 10·4 |
| Social Function | 1.11 | 18·5 | 1.03 | 24·7 |
| Fatigue | 1.03 | -14·5 | 0.96 | -21·9 |
| Nausea/vomiting | 0.83 | -8·1 | 0.86 | -12·2 |
| Pain | 1.02 | -11·2 | 0.98 | -17·5 |
| Dyspnea | 1.09 | -10·0 | 1.07 | -15·3 |
| Insomnia | 1.15 | -16·1 | 1.06 | -16·9 |
| Appetite loss | 1.21 | -18·9 | 1.17 | -22·9 |
| Constipation | 1.02 | -13·6 | 0.97 | -16·2 |
| Diarrhea | 0.80 | -4·2 | 0.79 | -6·4 |
| Financial Problems | 1.14 | -21·0 | 1.06 | -25·6 |

GP/ND/Rec General Population /Newly Diagnosed/Recurrent
