# Supplementary material for: Can Quality of Life Assessments Differentiate Heterogeneous Cancer Patients?
Source: PLoS One. 2014 Jun 11;9(6):e99445. doi: 10.1371/journal.pone.0099445 (PMC4053440; doi:10.1371/journal.pone.0099445)
Supplement: File S1 — Contains the files: Table S1- Mean, median and standard deviations of QoL attributes for EORTC general population (7802), newly diagnosed (3775) and recurrent disease (4711) patients. Table S2- Mean, median and standard deviation of QoL attributes of patients with respect to Mortality < = 3-months Vs >3-months. Table S3- Mean, median and standard deviation of QoL attributes of patients with respect to Stage 1&2 vs 3&4. Table S4- Mean, median and standard deviation of QoL attributes of patients with respect to Comorbidities <3 vs > = 3. Table S5- Mean, median and standard deviation of QoL attributes of patients with respect to Gender and class of case. Table S6- Mean, median and standard deviation of QoL attributes of patients with respect to median Age and class of case. Table S7- Comparison of mean scores between EORTC published general population and newly diagnosed patients with early stage disease. Table S8- Confidence intervals of Patient sub-groups by Site of Origin. Table S9- Confidence intervals for EORTC General Population compared with newly diagnosed and recurrent patients. Table S10- QoL scale scores and differences between patient sub-groups by site of origin. Table S11- Summary of sub-group comparisons within population, disease severity and demographic characteristics. (ZIP) [file pone.0099445.s001.zip › Table S8.docx]

Table S8: Confidence intervals of Patient sub-groups by Site of Origin

| QOL symptoms  and functions | Prostate | | Breast | | Colorectal | | Lung | | Pancreatic | | Others | |
| --- | --- | --- | --- | --- | --- | --- | --- | --- | --- | --- | --- | --- |
|  | CI 95% (±) | QoL Diff | CI 95% (±) | QoL Diff | CI 95% (±) | QoL Diff | CI 95% (±) | QoL Diff | CI 95% (±) | QoL Diff | CI 95% (±) | QoL Diff |
| Global Health | 3.64 | 12·6 | 2.38 | 9·4 | 3.61 | 5·1 | 2.73 | 6·0 | 3.70 | 2·4***** | 1.93 | 3·4 |
| Physical Function | 2.97 | 10·9 | 2.13 | 12·6 | 3.47 | 6·4 | 2.60 | 9·0 | 3.38 | 4·7 | 1.80 | 4·8 |
| Role Function | 4.10 | 13·6 | 2.99 | 11·8 | 4.82 | 1·9***** | 3.59 | 6·5 | 5.01 | 1·1***** | 2.50 | 2·0**^§^** |
| Emotional Function | 3.42 | 5·3 | 2.34 | -1·1***** | 3.59 | 1·1***** | 2.72 | -1·5***** | 3.63 | -3·7**^§^** | 1.83 | -2·5 |
| Cognitive Function | 3.02 | 7·3 | 2.32 | 3·1**^§^** | 3.48 | 3·3***** | 2.73 | 3·3**^§^** | 3.65 | -1·0***** | 1.89 | 0·4***** |
| Social Function | 4.00 | 12·5 | 2.94 | 10·1 | 4.65 | 5·1**^§^** | 3.45 | 7·7 | 4.74 | -1·8***** | 2.44 | 2·6**^§^** |
| Fatigue | 3.73 | -12·5 | 2.57 | -11·9 | 4.17 | -5·7**^§^** | 2.94 | -7·4 | 4.11 | -3·0***** | 2.12 | -3·9 |
| Nausea/vomiting | 2.28 | -5·0**^§^** | 1.94 | -6·3 | 3.21 | -2·9**^§^** | 2.34 | -4·9 | 3.66 | 1·6***** | 1.76 | -3·4 |
| Pain | 4.22 | -15·7 | 2.91 | -12·2 | 4.68 | -6·0**^§^** | 3.48 | -6·2 | 4.79 | 3·0***** | 2.40 | -2·5**^§^** |
| Dyspnea | 3.24 | -5·6 | 2.65 | -10·9 | 4.04 | -4·5***** | 3.54 | -4·6**^§^** | 4.06 | -4·1***** | 2.18 | -3·6 |
| Insomnia | 4.32 | -6·0**^§^** | 3.12 | -2·6***** | 4.74 | -0·5***** | 3.53 | 1·6***** | 4.58 | 2·7***** | 2.41 | 1·1***** |
| Appetite loss | 3.48 | -11·1 | 2.83 | -7·8 | 4.77 | -3·9***** | 3.57 | -6·1**^§^** | 5.25 | 8·1 | 2.52 | -1·5***** |
| Constipation | 3.41 | -10·1 | 2.70 | -7·2 | 4.44 | 1·7***** | 3.33 | -2·6***** | 5.02 | 8·6 | 2.24 | -1·7***** |
| Diarrhea | 2.63 | -2·2***** | 1.98 | 0·0***** | 3.82 | 0·0***** | 2.04 | -1·3***** | 4.00 | -2·5***** | 1.79 | -3·3 |
| Financial Problems | 4.34 | -6·4**^§^** | 3.23 | -6·5 | 5.04 | -2·0***** | 3.61 | -5·0**^§^** | 5.01 | 4·3***** | 2.52 | -2·7**^§^** |

* Not Statistically Significant (p>0·05)

**^§^** Not Statistically Significant, multiple testing adjusted (p>0·0033)
