# Supplementary material for: Can Quality of Life Assessments Differentiate Heterogeneous Cancer Patients?
Source: PLoS One. 2014 Jun 11;9(6):e99445. doi: 10.1371/journal.pone.0099445 (PMC4053440; doi:10.1371/journal.pone.0099445)
Supplement: File S1 — Contains the files: Table S1- Mean, median and standard deviations of QoL attributes for EORTC general population (7802), newly diagnosed (3775) and recurrent disease (4711) patients. Table S2- Mean, median and standard deviation of QoL attributes of patients with respect to Mortality < = 3-months Vs >3-months. Table S3- Mean, median and standard deviation of QoL attributes of patients with respect to Stage 1&2 vs 3&4. Table S4- Mean, median and standard deviation of QoL attributes of patients with respect to Comorbidities <3 vs > = 3. Table S5- Mean, median and standard deviation of QoL attributes of patients with respect to Gender and class of case. Table S6- Mean, median and standard deviation of QoL attributes of patients with respect to median Age and class of case. Table S7- Comparison of mean scores between EORTC published general population and newly diagnosed patients with early stage disease. Table S8- Confidence intervals of Patient sub-groups by Site of Origin. Table S9- Confidence intervals for EORTC General Population compared with newly diagnosed and recurrent patients. Table S10- QoL scale scores and differences between patient sub-groups by site of origin. Table S11- Summary of sub-group comparisons within population, disease severity and demographic characteristics. (ZIP) [file pone.0099445.s001.zip › Table S7.docx]

Table S7: Comparison of mean scores between EORTC published general population and newly diagnosed patients with early stage disease.

| QoL Symptom and Function scales | Mean scores | | | | Differences between mean scores & CI 95% (±) | | | | | |
| --- | --- | --- | --- | --- | --- | --- | --- | --- | --- | --- |
|  | General Population | Newly diagnosed Stage 1&2 | Breast & Prostate ND Stage 1&2 | Other Sites ND stage 1&2 | GP - ND stage 1&2 | | GP - Breast & Prostate ND stage 1&2 | | GP - Other sites ND stage 1&2 | |
|  | N=7802 | N=1378 | N=888 | N=490 | CI 95% (±) | QoL diff | CI 95% (±) | QoL diff | CI 95% (±) | QoL diff |
| Global Health | 71.2 | 67.6 | 70.9 | 61.8 | 1.40 | 3.6 | 1.67 | 0.3 | 2.23 | 9.4 |
| Physical Function | 89.8 | 85.0 | 87.4 | 80.6 | 1.14 | 4.8 | 1.28 | 2.4 | 1.96 | 9.2 |
| Role Function | 84.7 | 77.9 | 82.3 | 70.0 | 1.61 | 6.8 | 1.82 | 2.4 | 2.91 | 14.7 |
| Emotional Function | 76.3 | 68.4 | 70.5 | 64.5 | 1.42 | 7.9 | 1.70 | 5.8 | 2.28 | 11.8 |
| Cognitive Function | 86.1 | 79.8 | 81.6 | 76.5 | 1.35 | 6.3 | 1.59 | 4.5 | 2.24 | 9.6 |
| Social Function | 87.5 | 76.7 | 80.6 | 69.7 | 1.63 | 10.8 | 1.87 | 6.9 | 2.87 | 17.8 |
| Fatigue | 24.1 | 30.7 | 26.9 | 37.8 | 1.48 | 6.6 | 1.75 | 2.8 | 2.42 | 13.7 |
| Nausea/vomiting | 3.7 | 7.6 | 6.3 | 10.0 | 0.98 | 3.9 | 1.07 | 2.6 | 1.64 | 6.3 |
| Pain | 20.9 | 24.3 | 20.8 | 30.6 | 1.48 | 3.4 | 1.71 | -0.1 | 2.66 | 9.7 |
| Dyspnea | 11.8 | 16.5 | 13.3 | 22.2 | 1.47 | 4.7 | 1.60 | 1.5 | 2.62 | 10.4 |
| Insomnia | 21.8 | 34.3 | 31.8 | 38.8 | 1.75 | 12.5 | 2.14 | 10.0 | 2.82 | 17.0 |
| Appetite loss | 6.7 | 16.4 | 13.1 | 22.2 | 1.55 | 9.7 | 1.70 | 6.4 | 2.73 | 15.5 |
| Constipation | 6.7 | 14.9 | 12.3 | 19.5 | 1.38 | 8.2 | 1.50 | 5.6 | 2.62 | 12.8 |
| Diarrhea | 7.0 | 10.7 | 9.2 | 13.3 | 1.18 | 3.7 | 1.32 | 2.2 | 2.12 | 6.3 |
| Financial Problems | 9.5 | 26.6 | 23.7 | 31.8 | 1.77 | 17.1 | 2.12 | 14.2 | 3.05 | 22.3 |


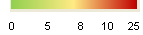
 Clinical relevance based on magnitude of point difference (Small: 5-10, Moderate: 10-20, Large: >20)

GP/ND General Population /Newly Diagnosed
