# Supplementary material for: Can Quality of Life Assessments Differentiate Heterogeneous Cancer Patients?
Source: PLoS One. 2014 Jun 11;9(6):e99445. doi: 10.1371/journal.pone.0099445 (PMC4053440; doi:10.1371/journal.pone.0099445)
Supplement: File S1 — Contains the files: Table S1- Mean, median and standard deviations of QoL attributes for EORTC general population (7802), newly diagnosed (3775) and recurrent disease (4711) patients. Table S2- Mean, median and standard deviation of QoL attributes of patients with respect to Mortality < = 3-months Vs >3-months. Table S3- Mean, median and standard deviation of QoL attributes of patients with respect to Stage 1&2 vs 3&4. Table S4- Mean, median and standard deviation of QoL attributes of patients with respect to Comorbidities <3 vs > = 3. Table S5- Mean, median and standard deviation of QoL attributes of patients with respect to Gender and class of case. Table S6- Mean, median and standard deviation of QoL attributes of patients with respect to median Age and class of case. Table S7- Comparison of mean scores between EORTC published general population and newly diagnosed patients with early stage disease. Table S8- Confidence intervals of Patient sub-groups by Site of Origin. Table S9- Confidence intervals for EORTC General Population compared with newly diagnosed and recurrent patients. Table S10- QoL scale scores and differences between patient sub-groups by site of origin. Table S11- Summary of sub-group comparisons within population, disease severity and demographic characteristics. (ZIP) [file pone.0099445.s001.zip › Table S6.docx]

Table S6: Mean, median and standard deviation of QoL attributes of patients with respect to median Age and class of case

| QOL symptoms  and functions | Newly Diagnosed Age<57 | | | p-values (${\mathrm{Mann}-Whitney test}^{*}$) | CI 95% (±) | Quality of Life Differences | Newly Diagnosed Age>=57 | | | Recurrent Age<55 | | | p-values (${\mathrm{Mann}-Whitney test}^{*}$) | CI 95% (±) | Quality of Life Differences | Recurrent Age>=55 | | |
| --- | --- | --- | --- | --- | --- | --- | --- | --- | --- | --- | --- | --- | --- | --- | --- | --- | --- | --- |
|  | 1830 | | |  |  |  | 1937 | | | 2180 | | |  |  |  | 2531 | | |
|  | Mean | Median | Standard Deviation |  |  |  | Mean | Median | Standard Deviation | Mean | Median | Standard Deviation |  |  |  | Mean | Median | Standard Deviation |
| Global Health | 61.4 | 66.7 | 25.6 | 0.4858 | 1.65 | 0.1 | 61.3 | 66.7 | 26.1 | 56.2 | 58.3 | 25.5 | 0.0676 | 1.48 | 1.4 | 54.8 | 58.3 | 26.2 |
| Physical Function | 80.8 | 86.7 | 22.1 | <0.0001 | 1.43 | 2.9 | 77.9 | 86.7 | 22.7 | 72.8 | 80.0 | 24.2 | 0.0002 | 1.42 | 2.7 | 70.1 | 73.3 | 25.3 |
| Role Function | 68.3 | 66.7 | 33.1 | 0.1158 | 2.10 | -1.3 | 69.6 | 83.3 | 32.8 | 62.0 | 66.7 | 33.5 | 0.0442 | 1.93 | -1.5 | 63.5 | 66.7 | 33.8 |
| Emotional Function | 62.5 | 66.7 | 25.3 | <0.0001 | 1.59 | -6.2 | 68.7 | 75.0 | 24.5 | 64.2 | 66.7 | 25.1 | <0.0001 | 1.42 | -4.1 | 68.3 | 75.0 | 24.4 |
| Cognitive Function | 76.5 | 83.3 | 25.1 | <0.0001 | 1.55 | -3.4 | 79.9 | 83.3 | 23.3 | 74.6 | 83.3 | 25.6 | 0.0027 | 1.44 | -2.1 | 76.7 | 83.3 | 24.7 |
| Social Function | 67.5 | 66.7 | 31.9 | 0.0009 | 2.03 | -3.0 | 70.5 | 83.3 | 31.6 | 60.8 | 66.7 | 32.5 | <0.0001 | 1.86 | -3.8 | 64.6 | 66.7 | 32.5 |
| Fatigue | 39.5 | 33.3 | 28.2 | 0.0292 | 1.79 | 1.7 | 37.8 | 33.3 | 28.0 | 46.5 | 44.4 | 28.6 | 0.1858 | 1.64 | 0.9 | 45.6 | 44.4 | 28.6 |
| Nausea/vomiting | 13.3 | 0.0 | 20.6 | <0.0001 | 1.27 | 2.9 | 10.4 | 0.0 | 19.2 | 17.8 | 0.0 | 25.1 | <0.0001 | 1.36 | 3.5 | 14.3 | 0.0 | 22.5 |
| Pain | 34.5 | 33.3 | 31.4 | <0.0001 | 1.98 | 4.8 | 29.7 | 16.7 | 30.4 | 40.6 | 33.3 | 33.0 | <0.0001 | 1.88 | 4.2 | 36.4 | 33.3 | 32.7 |
| Dyspnea | 20.2 | 0.0 | 27.3 | 0.0034 | 1.81 | -3.0 | 23.2 | 0.0 | 29.3 | 26.5 | 33.3 | 30.6 | 0.1099 | 1.76 | -1.2 | 27.7 | 33.3 | 31.0 |
| Insomnia | 40.9 | 33.3 | 32.5 | <0.0001 | 2.05 | 5.8 | 35.1 | 33.3 | 31.8 | 42.3 | 33.3 | 33.0 | <0.0001 | 1.86 | 6.6 | 35.7 | 33.3 | 32.1 |
| Appetite loss | 27.1 | 33.3 | 31.9 | 0.0006 | 2.04 | 3.0 | 24.1 | 0.0 | 31.9 | 30.2 | 33.3 | 33.5 | 0.07 | 1.93 | 1.1 | 29.1 | 33.3 | 33.9 |
| Constipation | 20.4 | 0.0 | 29.2 | 0.3585 | 1.87 | 0.2 | 20.2 | 0.0 | 29.2 | 24.4 | 0.0 | 31.7 | 0.0083 | 1.76 | 2.8 | 21.6 | 0.0 | 29.7 |
| Diarrhea | 12.5 | 0.0 | 23.1 | 0.0068 | 1.38 | 2.6 | 9.9 | 0.0 | 20.1 | 14.1 | 0.0 | 24.3 | 0.1158 | 1.35 | 1.2 | 12.9 | 0.0 | 22.8 |
| Financial Problems | 34.8 | 33.3 | 35.0 | <0.0001 | 2.12 | 8.4 | 26.4 | 33.3 | 31.5 | 40.5 | 33.3 | 35.2 | <0.0001 | 1.94 | 10.1 | 30.4 | 33.3 | 32.6 |

* Mann-Whitney test, also known as rank sum test, is a non-parametric test that compares two independent groups.
